# Phytochemistry, bioactivity, and *in silico* studies on two understudied *Achillea* species (*A. goniocephala* and *A. brachyphylla*): species- and solvent-dependent patterns

**DOI:** 10.1039/d6ra08354d

**Published:** 2026-10-02

**Authors:** Milena Terzic, Alina Kalyniukova, Vasil Andruch, Mehmet Veysi Cetiz, Abdullahi Ibrahim Uba, Nadire Pelin Bahadırlı, Mehmet Maruf Balos, Gunes Ak, Enver Saka, Sakina Yagi, Gokhan Zengin

**Affiliations:** a Faculty of Technology Novi Sad, University of Novi Sad Novi Sad Serbia; b Faculty of Forestry and Wood Sciences, Czech University of Life Sciences Prague CZ-165 21 Praha 6 Suchdol Czech Republic; c Institute of Chemistry, Faculty of Science, Pavol Jozef Šafárik University in Košice SK-041 54 Košice Slovakia; d Department of Medical Biochemistry, Faculty of Medicine, Harran University Sanliurfa 63290 Türkiye; e Department of Biostatistics and Medical Informatics, Faculty of Medicine, İstinye University 34396 Istanbul Türkiye abdullahi.uba@istinye.edu.tr; f Department of Field Crop, Faculty of Agriculture, Hatay Mustafa Kemal University 31060 Hatay Türkiye; g Şanlıurfa Provincial Directorate of National Education, Mehmet Gunes Anatolian High School TR-63000 Haliliye Şanlıurfa Turkey; h Department of Biology, Faculty of Science, Selcuk University Konya Turkey; i Department of Biology, Faculty of Science, Khartoum University Khartoum Sudan

## Abstract

The solvent-dependent phytochemical and biological profiles of *Achillea goniocephala* and *Achillea brachyphylla* remain poorly characterised. Five aerial-part extracts in solvents of increasing polarity (*n*-hexane, ethyl acetate, ethanol, ethanol/water and water) were analysed by LC-MS-QTOF, multivariate analysis, total phenolic and flavonoid assays, six antioxidant assays and five enzyme inhibition assays. More than 90 compounds, including acylquinic and phenolic acids, flavone *C*- and *O*-glycosides, methoxylated flavones and terpenoid-related metabolites, were tentatively annotated across negative and positive ionisation modes. PCA differentiated the species – extract combinations, with the first two components explaining 76.2% and 83.6% of the variance in negative and positive ionisation modes, respectively, while PERMANOVA confirmed significant overall differences (*p* = 0.001). Species-associated qualitative patterns were also evident: achillin/matricin and achimilic acid were characteristic of *A. goniocephala*, whereas deoxyloganin and verbascoside were associated with *A. brachyphylla*. The highest TPC was observed in the *A. goniocephala* water extract (41.09 mg GAE per g). Polar *A. goniocephala* extracts showed strong reducing capacity, reaching 281.37 mg TE per g in CUPRAC and 182.74 mg TE per g in FRAP. Enzyme inhibition was target-dependent: *A. brachyphylla* showed pronounced BChE and α-amylase inhibition in ethyl acetate and *n*-hexane fractions, respectively, whereas *A. goniocephala* showed comparatively strong α-glucosidase inhibition in medium-to-polar extracts; ethanol and ethanol/water extracts of both species formed the highest activity group against tyrosinase. Molecular docking identified favourable interactions of selected phytochemicals with the investigated enzyme targets, while molecular dynamics simulations confirmed the stability of the representative ligand–protein complexes. Overall, the solvent-resolved approach revealed marked chemical and functional diversities between the taxa and among the extracts of the same species, demonstrating that neither phytochemical composition nor biological activity can be adequately represented by a single extraction system. This solvent-based comparison of the selected *Achillea* species based on phytochemical profiling, *in vitro* bioassays, and *in silico* analysis identifies these less-studied species as potential candidates for further investigation as sources of bioactive compounds.

## Abstract 2

This integrated analysis highlights two underexplored *Achillea* species as promising sources of bioactive compounds for future research. Graphical abstract image designed with Google Gemini.

## Graphical abstract

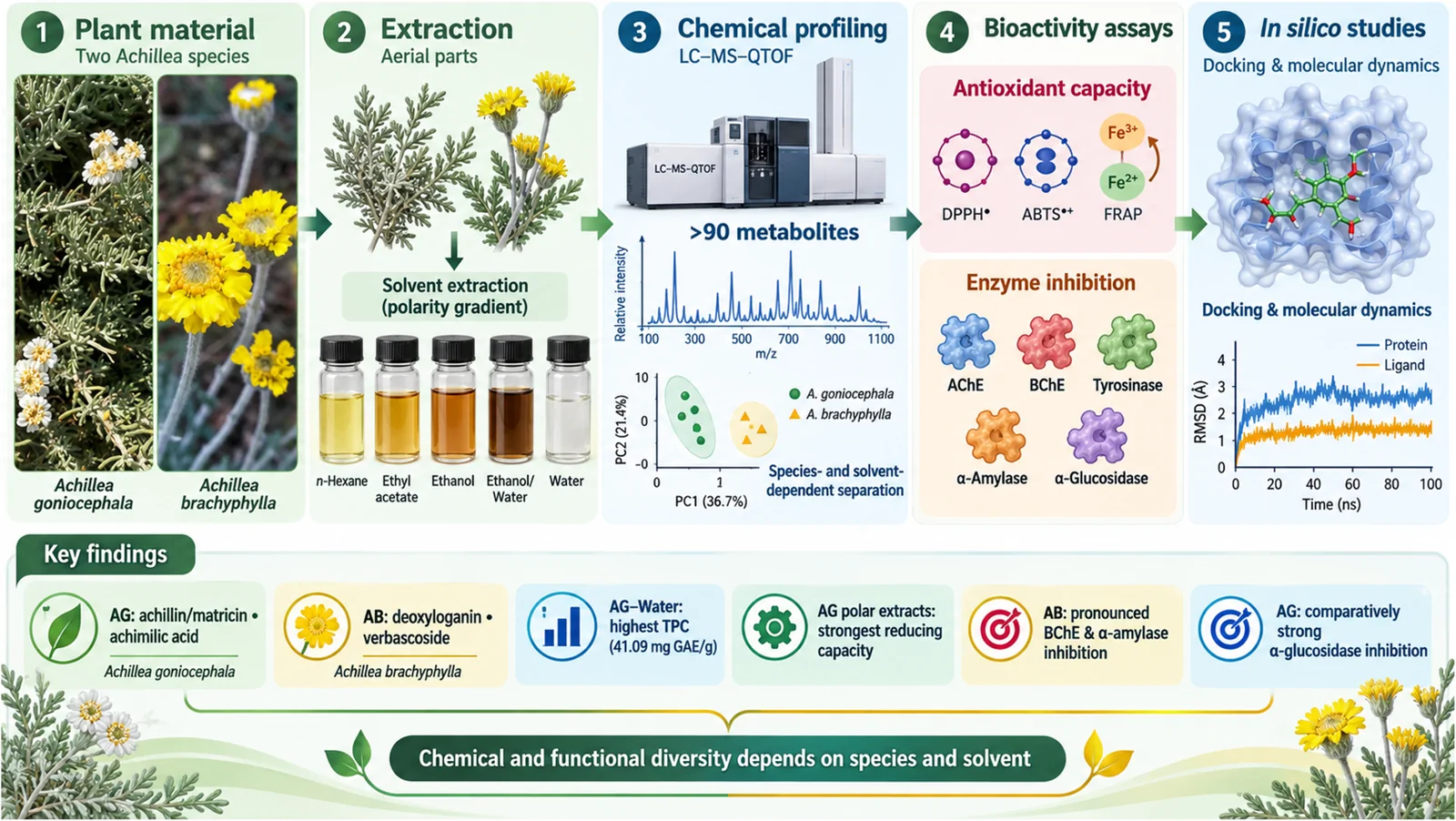

## Introduction

The genus *Achillea* L. (Asteraceae) comprises a taxonomically and chemically diverse group of medicinal plants with a long history of traditional use and considerable phytochemical properties. According to the recent literature, the genus *Achillea* comprises more than 130 species, predominantly distributed across the northern hemisphere, particularly in Europe and Asia. Türkiye is home to 47 *Achillea* species, 24 of which are endemic to the country.^1–3^ The genus *Achillea* is characterized by its remarkable adaptability to diverse environmental conditions, ranging from arid deserts and high-altitude mountainous regions to temperate agricultural fields.^1^ The genus also has a notable mythological background. According to accounts derived from Homer's *Iliad*, Achilles is believed to have used these plants to stop the bleeding and heal the wounds of his soldiers after battles.^1^ Research on the biological activities and traditional uses of *Achillea* species has provided valuable evidence regarding their noteworthy wound-healing and antimicrobial effects and anxiolytic, antibacterial, antifungal, anti-inflammatory, anthelmintic, anticholinesterase, antinociceptive, antioxidant, anti-arrhythmic, antitumor, antiulcer, anti-thrombotic, vasorelaxant, antiviral, skin-rejuvenating, diuretic, hemostatic, hepatoprotective, hypoglycemic, anti-spermatogenic, hypotensive, insecticidal, immunosuppressive, choleretic, estrogenic, spasmolytic, vasoprotective and gastroprotective properties.^1,3^ Previous investigations have shown that *Achillea* species contain a broad spectrum of specialized metabolites, including phenolic acids, particularly hydroxycinnamic and acylquinic acid derivatives, flavonoid glycosides and aglycones, methoxylated flavones, and terpenoid-related compounds such as sesquiterpene lactones. The qualitative and quantitative distributions of these constituents may vary considerably among species and even among different populations or plant materials of the same species, which is reflected in marked differences in their antioxidant, enzyme inhibitory and other biological properties. Consequently, the phytochemical and functional profiles of *Achillea* species are strongly influenced by taxonomic and biological variability, requiring evaluation at the level of individual taxa.^4–6^

Comparative investigations have provided particularly strong evidence for this diversity. Wild and commercial samples of *A. millefolium*, for example, displayed broadly similar qualitative phenolic profiles but differed considerably in the amounts of individual constituents and showed heterogeneous biological responses.^4^ Similarly, comparative analyses of several *Achillea* species have revealed pronounced interspecific differences in their phytochemical composition and biological activity.^5–8^ These findings emphasise the importance of species-specific characterisation, particularly for taxa for which chemical and functional information remains limited.

Extraction conditions represent an additional source of variation because the chemical and functional profiles of an extract reflect the fraction of the plant metabolome recovered under the selected conditions. Studies using solvents of contrasting polarity have demonstrated substantial differences in the composition and biological properties of *Achillea* extracts.^5,9–11^ In *A. aleppica* and *A. santolinoides*, different solvent systems yielded chemically distinct extracts with marked differences in phenolic composition and bioactivity,^9^ while similar solvent-dependent patterns have been reported for *A. phrygia*.^10^ More recently, the extraction of *A. maritima* across a broad polarity range has produced clearly differentiated polar and non-polar phytochemical profiles.^11^ Collectively, these observations indicate that the examination of a single extract may capture only part of the chemical and functional diversities of a species, an important consideration when poorly characterised taxa are investigated.

Despite the growing body of research on the genus, current knowledge remains unevenly distributed among individual *Achillea* taxa. *Achillea goniocephala* Boiss. & Balansa has previously been included in a comparative investigation of twelve Anatolian *Achillea* species, in which its phytochemical fingerprint, phenolic and flavonoid contents and selected biological activities were evaluated.^12^ Its antioxidant and cytotoxic properties have also been investigated independently using fractionated extracts and nanochitosan formulations.^13^ In contrast, *A. brachyphylla* Boiss. & Hausskn. has been addressed primarily in taxonomic and nomenclatural studies,^14,15^ whereas dedicated investigations of its phytochemical composition and biological properties remain scarce. Consequently, the chemical diversity and functional properties of this species remain insufficiently characterised.

No previous study appears to have directly compared *A. goniocephala* and *A. brachyphylla* under the same experimental conditions within a solvent-resolved framework integrating comprehensive phytochemical profiling with a broad assessment of *in vitro* biological activities. In addition, molecular docking and molecular dynamics simulations were employed as complementary *in silico* approaches to examine the interactions and stability of selected phytochemicals with relevant enzyme targets, thereby providing a structural context for the observed enzyme inhibitory activities. The present study therefore aimed to characterise and compare the extracts of the two species obtained across a wide polarity range, with particular emphasis on their phytochemical profiles, total phenolic and flavonoid contents, antioxidant properties, and enzyme inhibitory activities. By resolving these characteristics at the level of individual extracts, the study sought to identify species- and extraction-dependent patterns and to further investigate the molecular basis of the observed bioactivities through molecular docking and molecular dynamics (MD) simulations of selected phytoconstituents against relevant enzyme targets. Molecular docking and MD simulations provide complementary computational approaches for elucidating the molecular basis of interactions between bioactive compounds and therapeutic targets. Molecular docking enables the prediction of ligand-binding modes and the estimation of relative binding affinities, thereby facilitating the identification and prioritization of potentially active phytochemicals.^16^ However, docking represents a relatively static description of molecular recognition and may not adequately capture the conformational flexibility of proteins and ligands.^17^ MD simulations can therefore provide additional insights into the temporal stability of ligand–target complexes, conformational changes, and key intermolecular interactions under dynamic conditions. The integration of docking with MD simulations can consequently strengthen the mechanistic interpretation of experimental enzyme-inhibition findings and help prioritize promising compounds for further experimental validation. The integrated experimental and computational approach was intended to identify potentially bioactive constituents, assess their target interactions and binding stability, and provide a foundation for more targeted mechanistic investigation of these comparatively understudied *Achillea* taxa.

The present study therefore aimed to characterise and compare extracts of the two *Achiella* species obtained across a wide polarity range, with particular emphasis on their phytochemical profiles, total phenolic and flavonoid contents, antioxidant properties and enzyme inhibitory activities.

## Materials and methods

### Plant collection

Plant samples were collected in 2023 from South Area of Turkey (*Achillea brachyphylla*: Tek Mountains, 640 m, Şanlıurfa; *Achiella goniocephala:* Belen, 1363 m, Hatay). Dr Nadire Pelin Bahadırlı and Dr Mehmet Maruf Balos officially carried out the botanical identification. After collection, the aerial parts were separated and air-dried at room temperature in a shaded, well-ventilated area. Once the plant material dried completely, it was ground into a fine powder. The powdered samples were stored in light-proof containers under constant conditions to keep them chemically stable and prevent spoilage.

### Plant extract preparation

Five solvents covering a broad range of polarities were used for extraction: *n*-hexane, ethyl acetate, ethanol, 70% aqueous ethanol, and water. For each solvent, 10 g of dried plant material was combined with 200 mL of solvent, though the extraction method varied by solvent type. For the four organic solvents, extraction was done by maceration—soaking the plant material at room temperature for 24 hours. The water extraction (10 g sample) instead used a 15-minute hot-water infusion for 15 min. Afterward, the organic extracts were concentrated by rotary evaporation under reduced pressure, while the aqueous extract was freeze-dried. All extracts were kept at 4 °C until analysis.

### Total phenolic and flavonoid contents

Total phenolic and total flavonoid contents of the extracts were determined using validated colorimetric methods, following established procedures.^18^ Calibration curves were plotted with gallic acid (for phenolics) and rutin (for flavonoids), and the results are expressed as gallic acid equivalents (GAE) and rutin equivalents (RE) per gram of extract.

### LC-MS-qTOF analysis

The LC-MS-qTOF analysis was performed using an Agilent 1290 Infinity II UHPLC system coupled to an Agilent 6546 QTOF mass spectrometer. Separation was carried out using an InfinityLab Poroshell 120 EC-C18 column (2 × 150 mm, 2.7 µm) with a mobile phase of water containing 0.1% formic acid (A) and methanol (B) at a flow rate of 0.5 mL min^−1^, a column temperature of 35 °C and an injection volume of 1 µL. The elution program set was: 0–4 min, 85%A/15% B; 4–7 min, 75%A/25%B; 7–9 min, 68% A/32% B; 9–16 min, 60% A/40% B; 16–22 min, 45% A/55% B; 22–30 min, 5% A/95% B. The system operated in the negative ionization mode with a mass range of 100–1000 *m*/*z*. MS parameters included a drying gas temperature of 160 °C, a sheath gas flow of 12 L min^−1^ at 400 °C, a capillary voltage of 5.0 kV, a nozzle voltage of 2.0 kV, a fragmentor voltage of 140 V and collision energies of 10, 20 and 40 eV. The collision energy providing the most informative and reproducible fragmentation pattern was used for data interpretation. MS/MS spectra were acquired over 50–800 *m*/*z*. Reference ions at *m*/*z* 112.98 and 966.0 were used for mass calibration.

Raw LC-MS data were processed using Agilent MassHunter Profinder 10.0, including molecular feature extraction and chromatographic alignment. Features were extracted over an *m*/*z* range of 100–1000 using a mass tolerance of 2 mDa and a retention time tolerance of ±0.25 min. Chromatographic alignment was performed using a minimum intensity threshold of 5000 counts and a maximum allowable retention time shift of 0.5 min. The detected compounds were annotated by comparing their accurate masses, retention times, isotopic patterns, and characteristic MS/MS fragmentation spectra with the data available in the METLIN database, our in-house spectral library, and previously published literature.

### Antioxidant assays

The antioxidant activity was assessed using a panel of *in vitro* assays following the protocol described in a previous study.^18^ Reducing power was measured by the FRAP and CUPRAC methods, while the radical scavenging capacity was evaluated using the DPPH and ABTS assays. The results from these four assays were converted to Trolox equivalents and expressed as mg TE per gram of dried extract. The total antioxidant capacity was also measured by the phosphomolybdenum (PBD) assay (mmol TE per g) and the metal-chelating activity expressed as mg EDTA equivalents per gram (mg EDTAE per g).

### Enzyme inhibitory activity assays

The extracts were evaluated for inhibitory activity against five key enzymes—acetylcholinesterase (AChE), butyrylcholinesterase (BChE), tyrosinase, α-amylase, and α-glucosidase—using established colorimetric methods.^18^ To allow comparison across samples, inhibition results were expressed relative to reference compounds: cholinesterase inhibition as mg galantamine equivalents per gram (mg GALAE per g), α-amylase and α-glucosidase inhibition as mmol acarbose equivalents per gram (mmol ACAE per g), and tyrosinase inhibition as mg kojic acid equivalents per gram (mg KAE per g).

### Molecular docking

The molecular structures of the predominant metabolites reported for *Achillea goniocephala* and *A. brachyphylla* were considered for *in silico* screening. The compounds for which unambiguous canonical SMILES strings could be obtained were retained for computational ligand preparation. Structural models of the selected protein targets were obtained from the Protein Data Bank.^19^ The individual protein structures, docking-site definitions, and corresponding search – space parameters are reported in Table S1.

Ligand conformations were generated directly from the corresponding SMILES representations with Gypsum-DL. Protonation and tautomeric states appropriate to near-physiological conditions (pH 7.4) were considered during this procedure. Three-dimensional molecular geometries were generated in SDF format and subsequently inspected using RDKit. Where compatible parameters were available, molecular geometries were relaxed with the MMFF94 force field for up to 2000 iterations. The energetically preferred valid conformer obtained from this procedure was used as the docking input. The resulting ligand structures were subsequently processed with Meeko to generate PDBQT files required by AutoDock Vina, including assignment of partial charges, atom types, hydrogen atoms, rotatable bonds, and torsional relationships.

Potential ligand-recognition cavities were identified independently using the POCASA v1.1 server, and the coordinates of the predicted pockets were used to position the docking search spaces. Molecular docking was conducted with AutoDock Vina v1.1.2^20^ using an exhaustiveness value of 32. Candidate poses were ranked according to their predicted binding scores, and the best-scoring geometrically plausible orientation for each protein–ligand combination was retained for subsequent structural assessment. Intermolecular contacts were characterized using the Protein–Ligand Interaction Profiler (PLIP),^21^ including hydrogen bonding, hydrophobic interactions, aromatic contacts, salt bridges, and other non-covalent interactions identified within the predicted binding site. The highest-ranked complexes for each target were subsequently carried forward to molecular dynamics simulations.

### Molecular dynamics simulations

The selected docking poses (C1: orientin–BChE; C2: 3,5-diferuloylquinic acid–BChE; C3: orientin–AChE; C4: 3,5-diferuloylquinic acid–amylase; and C5: orientin–glucosidase) were used as starting configurations for explicit-solvent atomistic MD simulations. System construction was performed through the CHARMM-GUI platform,^22^ with the CHARMM36m force field adopted for the protein and ligand components. Each molecular system was embedded in an explicit TIP3P water environment and electrically neutralized with counterions. NaCl was then introduced to reproduce an ionic concentration of 0.15 M.

Periodic boundary conditions were applied throughout the simulations. Non-bonded interactions within the short-range cutoff were evaluated using the Verlet scheme, whereas electrostatic interactions beyond the short-range region were treated using particle-mesh Ewald (PME). The LINCS algorithm was used to constrain covalent bonds involving hydrogen atoms. Before thermal equilibration, each system was subjected to steepest-descent minimization to eliminate unfavorable steric contacts and high-energy geometries. Minimization was continued until the maximum force was reduced below 1000 kJ mol^−1^ nm^−1^.^23,24^

The equilibrated systems were subsequently subjected to sequential constant-volume (NVT) and constant-pressure (NPT) equilibration at 310 K. Production trajectories were then generated for 100 ns using GROMACS 2023.^25,26^ Trajectory processing was performed before quantitative analysis to ensure consistent treatment of periodic boundary effects and molecular coordinates. Structural and interactional properties were evaluated throughout the production trajectories using complementary descriptors. Global conformational behavior was monitored using RMSD, whereas residue-specific mobility was quantified using RMSF. The changes in solvent exposure were assessed from SASA profiles, while hydrogen bond analysis and protein–ligand contact monitoring were used to characterize the persistence and remodeling of intermolecular interactions. In addition, principal component analysis (PCA) was applied to identify the dominant collective motions sampled by each complex, and the corresponding free-energy landscapes (FELs) were generated to visualize the principal conformational states and transitions explored during the simulations.

### Statistical analysis

All experiments were conducted in triplicate. Statistical significance of variations among extract samples was assessed using one-way ANOVA in GraphPad Prism (version 9.2), followed by Tukey's post hoc test for pairwise comparisons. A *p*-value below 0.05 was considered statistically significant. Statistical analyses of LC-MS-QTOF data were conducted using the MetaboAnalyst platform (https://www.metaboanalyst.ca/). PCA was initially performed to visualize the overall metabolic variation and sample clustering. Partial least squares discriminant analysis (PLS-DA) was subsequently applied to assess group separation and identify features contributing to sample discrimination. Variable importance in projection (VIP) scores were calculated to determine the most discriminative features in both positive and negative ionization modes.

## Results and discussion

### Total phenolic (TPC) and flavonoid (TFC) contents

The total phenolic (TPC) and flavonoid contents (TFC) varied markedly among the extracts of both *Achillea* species (Table 1), revealing distinct responses to the extraction solvent. In *A. goniocephala*, the highest TPC values were recorded in the water (41.09 mg GAE per g) and ethanol/water (40.07 mg GAE per g) extracts, with no significant difference between them (*p* > 0.05). Both extracts contained significantly higher TPC than the ethanol, ethyl acetate and *n*-hexane extracts (*p* ≤ 0.05), whereas *n*-hexane yielded the lowest value (13.92 mg GAE per g). A different pattern was observed for *A. brachyphylla*: the ethanol extract showed the highest TPC (28.63 mg GAE per g) and differed significantly from all other extracts (*p* ≤ 0.05), while the ethanol/water and ethyl acetate extracts were statistically comparable. Thus, phenolic recovery in *A. goniocephala* was favoured by the most polar extraction systems, whereas *A. brachyphylla* exhibited a less monotonic response, with ethanol providing the highest TPC.

**Table 1:** Extraction yields (%) and total phenolic and flavonoid contents of the tested extracts^a^

Species	Extracts	Extraction yields (%)	TPC^b^ (mg GAE per g)	TFC^c^ (mg RE per g)
*A. brachyphylla*	*n*-Hexane	0.82	16.96 ± 0.42^f^	3.09 ± 0.12^h^
	EA	1.70	24.93 ± 0.69^d^	23.51 ± 0.13^d^
	Ethanol	2.58	28.63 ± 0.21^c^	46.30 ± 0.33^a^
	Ethanol/water	6.70	26.33 ± 0.42^d^	14.49 ± 0.13^g^
	Water	4.83	22.80 ± 0.36^e^	0.38 ± 0.02^i^
*A. goniocephala*	*n*-Hexane	1.77	13.92 ± 0.97^g^	21.96 ± 0.29^e^
	EA	3.98	16.79 ± 0.75^f^	32.94 ± 0.52^c^
	Ethanol	6.60	32.40 ± 0.95^b^	32.83 ± 0.24^c^
	Ethanol/water	15.30	40.07 ± 0.47^a^	35.43 ± 0.34^b^
	Water	8.70	41.09 ± 0.47^a^	17.38 ± 0.26^f^

a The values are presented as mean ± SD of three measurements. Means within each column with different letters (a–i) differ significantly (*p* ≤ 0.05).

b mg gallic acid equivalents per gram of extract.

c mg rutin equivalents per gram of extract.

A comparable preference for polar extraction systems has been reported for *A. maritima*, in which the water extract displayed the highest TPC (32.26 mg GAE per g), followed by ethanol/water (29.20 mg GAE per g), whereas the *n*-hexane extract contained the lowest level (19.28 mg GAE per g).^11^ Pronounced differences in phenolic recovery have also been reported for *A. aleppica* and *A. santolinoides*.^9^ In that study, the highest TPC for *A. aleppica* was observed in the aqueous root extract (43.24 mg GAE per g), whereas the methanolic root extract of *A. santolinoides* contained the highest TPC within that species (52.07 mg GAE per g).^9^ Together with the present findings, these results indicate that the extraction behaviour of phenolic constituents cannot be explained by solvent polarity alone, but also depends on the species-specific composition of the plant matrix. Direct numerical comparisons among studies should nevertheless be made cautiously because the investigated plant material and extraction conditions are not identical.

The TFC results showed a clearly different distribution from the TPC. In *A. brachyphylla*, ethanol gave the highest TFC by a substantial margin (46.30 mg RE per g), significantly exceeding all other extracts (*p* ≤ 0.05), whereas the water extract contained only 0.38 mg RE per g. In *A. goniocephala*, the ethanol/water extract exhibited the highest TFC (35.43 mg RE per g), followed by ethyl acetate and ethanol (32.94 and 32.83 mg RE per g, respectively), with no significant difference between the latter two extracts (*p* > 0.05). The contrasting behaviour of the aqueous extracts is particularly notable: water yielded the highest TPC in *A. goniocephala* but a substantially lower TFC, while the aqueous extract of *A. brachyphylla* retained an appreciable TPC despite showing almost negligible TFC. These findings indicate that the compounds contributing to the overall TPC response were distributed differently from those quantified by the flavonoid assay.

A similar divergence between TPC and TFC has been observed in other *Achillea* species. In *A. maritima*, although water gave the highest TPC, the ethyl acetate extract contained the highest TFC (7.83 mg RE per g), whereas the aqueous extract contained only 2.83 mg RE per g.^8^ Similarly, in *A. aleppica*, the methanolic aerial-part extract showed the highest TFC (49.18 mg RE per g), whereas the highest TPC was found in the aqueous root extract.^9^ The exceptionally high TFC of the *A. brachyphylla* ethanol extract observed here is therefore consistent with the preferential recovery of flavonoid-reactive constituents by this solvent rather than with a general increase in total phenolic response.

The interpretation of TPC should also take into account the analytical nature of the Folin–Ciocalteu assay. The reagent is not specific exclusively to phenolic compounds; systematic evaluation of more than 80 compounds demonstrated measurable reactivity with several non-phenolic reducing substances, including thiols, proteins, vitamins and selected inorganic species.^27^ Consequently, the particularly high TPC response of the aqueous and hydroalcoholic *A. goniocephala* extracts should not be interpreted as direct quantitative evidence for every individual phenolic class. Rather, the distinct TPC and TFC patterns provide an initial indication of substantial compositional differences among extracts, which can be examined more specifically using the LC-MS-QTOF profiles presented in the following section.

### LC-MS-QTOF profiling and multivariate analysis

LC-MS-QTOF profiling revealed a chemically complex metabolite pattern in both *A. goniocephala* and *A. brachyphylla*, with more than 90 compounds tentatively annotated across the negative and positive ionisation datasets (Table 2 and 3). The detected constituents encompassed several major phytochemical classes, including phenolic and organic acids, acylquinic acid derivatives, flavonoids, terpenoid-related metabolites, amino acids and fatty acid derivatives. Although the two species shared a substantial proportion of their chemical space, pronounced differences were evident among individual extracts, indicating considerable variation in metabolite composition associated with both plant identity and extraction conditions.

**Table 2:** List of tentatively identified compounds from *Achillea* extracts in the negative ionization mode^a^

Compounds	Mass	RT, min	ESI^−^	Formula	AG_H	AG_EA	AG_E	AG_E_W	AG_W	AB_H	AB_EA	AB_E	AB_E_W	AB_W	Ref.
Malic acid	134.0217	1.53	[M − H]^−^	C_4_H_6_O_5_	✓	✓	✓	✓	✓		✓	✓	✓	✓	8
Monosaccharide	150.053	1.45	[M − H]^−^	C_5_H_10_O_5_	✓		✓	✓	✓				✓	✓	
Protocatechuic acid	154.0267	5.16	[M − H]^−^	C_7_H_6_O_4_		✓	✓	✓	✓		✓	✓	✓	✓	8 and 9
Caffeic acid	180.0424	9.45	[M − H]^−^	C_9_H_8_O_4_		✓	✓	✓	✓		✓	✓	✓	✓	8 and 9
Disaccharide	180.0635	1.35	[M − H]^−^	C_6_H_12_O_6_	✓	✓		✓	✓		✓		✓		
Citric acid/isocitric acid	192.0271	1.90	[M − H]^−^	C_6_H_8_O_7_	✓	✓	✓	✓	✓		✓	✓	✓	✓	
Quinic acid	192.0635	1.37	[M − H]^−^	C_7_H_12_O_6_	✓	✓	✓	✓	✓		✓	✓	✓	✓	8 and 9
Achillin/matricin	246.1258	24.50	[M − H]^−^	C_15_H_18_O_3_	✓	✓	✓	✓	✓						9
Prenyl caffeate	248.1048	9.85	[M − H]^−^	C_14_H_16_O_4_	✓	✓	✓	✓	✓						
Hydroxyferulate	256.0584	4.97	[M − H]^−^	C_10_H_10_O_5_	✓	✓	✓	✓	✓						
Abscisic acid	264.1361	19.68	[M − H]^−^	C_15_H_20_O_4_		✓	✓	✓	✓		✓	✓	✓	✓	
Flavonol-*O*-D-xylosylglucoside	266.0793	6.32	[M − H]^−^		✓	✓	✓	✓	✓						
Achimilic acid	278.1156	13.23	[M − H]^−^	C_15_H_18_O_5_	✓	✓	✓	✓	✓						
Hydroxybenzoic acid-hexoside isomer 1	300.0635	24.82	[M − H]^−^					✓	✓		✓	✓	✓	✓	9
Hydroxybenzoic acid-hexoside isomer 2	300.0637	24.83	[M − H]^−^			✓	✓	✓	✓		✓	✓	✓	✓	9
Achillicin/matricin	308.1262	14.30	[M − H]^−^	C_17_H_22_O_5_	✓	✓	✓	✓	✓						9
Dihydroxy-dimethoxyflavone	314.079	25.61	[M − H]^−^	C_17_H_14_O_6_	✓	✓	✓	✓	✓	✓	✓	✓	✓	✓	
Protocatechuic acid-hexoside	316.0582	23.14	[M − H]^−^	C_13_H_16_O_9_	✓	✓	✓	✓	✓		✓	✓	✓	✓	9
*p*-Coumaric acid-hexoside	326.1006	13.57	[M − H]^−^	C_15_H_18_O_8_	✓	✓	✓	✓	✓						
Cirsiliol	330.074	24.92	[M − H]^−^	C_17_H_14_O_7_	✓	✓	✓	✓	✓	✓	✓	✓		✓	9
Vanillic acid-*O*-hexoside	330.1316	11.83	[M − H]^−^	C_14_H_18_O_9_			✓	✓	✓		✓	✓	✓	✓	9
*p*-Coumaroylquinic acid isomer 1	338.1002	10.37	[M − H]^−^	C_16_H_18_O_8_			✓	✓	✓		✓	✓	✓	✓	9
*p*-Coumaroylquinic acid isomer 2	338.1002	11.91	[M − H]^−^	C_16_H_18_O_8_	✓		✓	✓	✓			✓	✓	✓	9
Caffeic acid *O*-hexoside	342.0952	11.35	[M − H]^−^	C_15_H_18_O_9_			✓	✓	✓						9
Sucrose	342.1163	1.35	[M − H]^−^	C_12_H_22_O_11_	✓	✓	✓			✓	✓	✓	✓	✓	
Tetrahydroxy-dimethoxyflavone	346.0695	24.05	[M − H]^−^	C_17_H_14_O_8_							✓	✓	✓		
Chlorogenic acid isomer 1	354.0952	5.84	[M − H]^−^	C_16_H_18_O_9_			✓	✓	✓			✓	✓	✓	8, 9 and 29
Chlorogenic acid isomer 2	354.0953	9.07	[M − H]^−^	C_16_H_18_O_9_			✓	✓	✓			✓	✓	✓	9
Chlorogenic acid isomer 3	354.0955	8.52	[M − H]^−^	C_16_H_18_O_9_	✓	✓	✓	✓	✓		✓	✓	✓	✓	9
Syringic acid-*O*-hexoside	360.1057	6.45	[M − H]^−^	C_15_H_20_O_10_		✓	✓	✓	✓		✓	✓	✓	✓	9
Tetrahydroxy-methoxyflavanone	364.0797	11.73	[M − H]^−^	C_16_H_14_O_7_			✓	✓	✓						
*O*-Feruloylquinic acid	368.1107	10.98	[M − H]^−^	C_17_H_20_O_9_	✓	✓	✓	✓	✓		✓	✓	✓	✓	9
*O*-Caffeoyl-*O*-methylquinic acid	368.1109	8.56	[M − H]^−^	C_17_H_20_O_9_			✓	✓	✓			✓	✓	✓	
Dihydroferulic acid-*O*-glucuronide	372.1059	10.25	[M − H]^−^	C_16_H_20_O_10_			✓	✓	✓						
Deoxyloganin	374.1576	12.88	[M − H]^−^	C_16_H_24_O_9_							✓	✓	✓	✓	
Tuberonic acid glucoside	388.1734	9.91	[M − H]^−^	C_18_H_28_O_9_	✓	✓	✓	✓	✓						
Flavonoid hexoside	394.0901	12.26	[M − H]^−^		✓	✓	✓	✓	✓						
*O*-Caffeoylquinic acid	400.1007	8.16	[M − H]^−^	C_16_H_18_O_9_		✓	✓	✓	✓		✓	✓	✓	✓	
Tetrahydroxy-di-*C*-prenylflavanone	424.1865	12.58	[M − H]^−^	C_25_H_28_O_6_		✓	✓	✓	✓						
Vitexin/isovitexin	432.1054	18.15	[M − H]^−^	C_21_H_20_O_10_		✓	✓	✓	✓		✓	✓	✓		29
Isovitexin/vitexin	432.1058	15.45	[M − H]^−^	C_21_H_20_O_10_		✓	✓	✓	✓		✓	✓	✓	✓	29
Catechin-*O*-gallate	442.0899	22.90	[M − H]^−^	C_22_H_18_O_10_		✓	✓	✓	✓		✓	✓	✓		
Apigenin-*O*-pentoside	446.0847	18.09	[M − H]^−^	C_21_H_18_O_11_	✓		✓	✓	✓			✓	✓		
Apigenin-methyl ether-hexoside	446.1216	15.79	[M − H]^−^	C_22_H_22_O_10_		✓	✓	✓	✓						
Orientin	448.1007	12.79	[M − H]^−^	C_21_H_20_O_11_			✓	✓	✓			✓	✓		9 and 29
Luteolin-*O*-hexoside	448.1007	15.62	[M − H]^−^	C_21_H_20_O_11_	✓	✓	✓	✓	✓		✓	✓	✓	✓	9 and 29
Kaempferol O-hexoside	448.101	13.25	[M − H]^−^	C_21_H_20_O_11_	✓	✓	✓	✓	✓		✓	✓	✓	✓	9
Trihydroxy-methoxyflavone-*O*-glucoside isomer 1	462.1163	16.09	[M − H]^−^	C_22_H_22_O_11_		✓	✓	✓	✓		✓	✓	✓	✓	
Verbascoside	462.1736	9.36	[M − H]^−^	C_20_H_30_O_12_		✓	✓	✓	✓		✓	✓	✓	✓	
Quercetin hexoside isomer 1	464.0954	14.44	[M − H]^−^	C_21_H_20_O_12_	✓	✓	✓	✓	✓	✓					
Quercetin hexoside isomer 2	464.0955	16.45	[M − H]^−^	C_21_H_20_O_13_		✓	✓	✓	✓		✓	✓	✓	✓	
Quercetin hexoside isomer 3	464.0956	13.52	[M − H]^−^	C_21_H_20_O_14_			✓	✓	✓		✓	✓	✓		
Epicatechin-*O*-glucuronide	466.1114	8.76	[M − H]^−^	C_21_H_22_O_12_		✓	✓	✓	✓						
Vanillic acid-*O*-(-*O*-caffeoyl)-hexoside	492.1271	17.15	[M − H]^−^	C_23_H_24_O_12_		✓	✓	✓	✓			✓	✓		9
Di-*O*-caffeoylquinic acid isomer 1	516.1267	15.13	[M − H]^−^	C_25_H_24_O_12_	✓	✓	✓	✓	✓		✓	✓	✓	✓	9
Di-*O*-caffeoylquinic acid isomer 2	516.1269	18.02	[M − H]^−^	C_25_H_24_O_12_	✓	✓	✓	✓	✓		✓	✓	✓	✓	9
Di-*O*-caffeoylquinic acid isomer 3	516.127	14.81	[M − H]^−^	C_25_H_24_O_12_			✓	✓	✓			✓	✓	✓	9
Trihydroxy-trimethoxyisoflavone-*O*-glucoside	522.1373	14.15	[M − H]^−^	C_24_H_26_O_13_		✓	✓	✓	✓			✓	✓		
Feruloyl-5-caffeoylquinic acid isomer 1	530.1425	20.27	[M − H]^−^	C_26_H_26_O_12_	✓	✓	✓	✓	✓	✓	✓	✓	✓	✓	9
Feruloyl-5-caffeoylquinic acid isomer 2	530.1426	18.04	[M − H]^−^	C_26_H_26_O_13_		✓	✓	✓	✓		✓	✓	✓	✓	9
Diferuloylquinic acid	544.1578	22.22	[M − H]^−^	C_27_H_28_O_12_			✓	✓	✓		✓	✓	✓	✓	
Diferuloylquinic acid	544.1580	20.58	[M − H]^−^	C_27_H_28_O_12_		✓	✓	✓	✓		✓	✓	✓	✓	
*C*-Hexosyl-*C*-pentosyl apigenin 1	564.1476	12.61	[M − H]^−^	C_26_H_28_O_14_	✓	✓	✓	✓	✓				✓	✓	
*C*-Hexosyl-*C*-pentosyl apigenin 2	564.1479	14.19	[M − H]^−^	C_26_H_28_O_14_			✓	✓	✓		✓	✓	✓	✓	9
Isovitexin *O*-xyloside	564.148	13.06	[M − H]^−^	C_26_H_28_O_14_	✓	✓	✓	✓	✓		✓	✓	✓	✓	
Apigenin derivate	564.1484	14.66	[M − H]^−^	C_26_H_28_O_14_			✓	✓	✓		✓	✓	✓	✓	
Apigenin derivate 2	564.1487	15.88	[M − H]^−^	C_26_H_28_O_14_								✓	✓	✓	
Hydroxyapigenin-xylosyl-galactoside	580.1427	11.77	[M − H]^−^		✓	✓	✓	✓	✓			✓	✓	✓	
Hydroxyluteolin diglycoside	580.1429	11.60	[M − H]^−^	C_26_H_28_O_15_	✓	✓	✓	✓	✓	✓					
Quercetin-xyloside-rhamnoside	580.1429	13.76	[M − H]^−^	C_26_H_28_O_15_		✓	✓	✓	✓			✓	✓	✓	
*C*-Hexosyl-*C*-pentosylluteolin	580.1434	11.27	[M − H]^−^	C_26_H_28_O_15_			✓	✓	✓						
*o*-Pentosyl-*C*-hexosylmethylluteolin	594.1578	19.25	[M − H]^−^	C_27_H_30_O_15_		✓	✓	✓	✓			✓	✓		9
Orientin-*O*-rhamnoside	594.158	11.22	[M − H]^−^	C_27_H_30_O_15_			✓	✓	✓			✓	✓	✓	
Apigenin di-glucoside 1	594.1583	15.42	[M − H]^−^	C_27_H_30_O_15_		✓	✓	✓	✓		✓	✓	✓	✓	
Apigenin di-glucoside 2	594.1583	16.54	[M − H]^−^	C_27_H_30_O_15_		✓	✓	✓	✓		✓	✓	✓	✓	9 and 29
Luteolin 3′-methyl ether 7-arabinosyl-(1-2)-galactoside	594.1585	15.55	[M − H]^−^	C_28_H_32_O_16_		✓	✓	✓	✓						
Vitexin-*O*-hexoside	594.1588	13.87	[M − H]^−^	C_27_H_30_O_15_		✓	✓	✓	✓		✓	✓	✓	✓	
Rutin	610.1537	16.47	[M − H]^−^	C_27_H_30_O_16_	✓	✓	✓	✓	✓		✓	✓	✓		8 and 9
Luteolin dihexoside	610.1542	10.36	[M − H]^−^	C_27_H_30_O_16_			✓	✓	✓					✓	29
Isorhamnetin dihexoside	624.1686	10.85	[M − H]^−^	C_26_H_32_O_16_			✓	✓	✓			✓	✓	✓	
Verbascoside	624.205	14.55	[M − H]^−^	C_29_H_36_O_15_							✓	✓	✓		
Isovitexin dihexoside	726.2006	10.77	[M − H]^−^	C_27_H_30_O_15_			✓	✓	✓			✓	✓	✓	
Naringin rhamnoside	726.2371	23.39	[M − H]^−^	C_33_H_42_O_18_							✓	✓	✓	✓	
Isorientin-*O*-glucoside-*O*-hydroxybenzoate	730.1723	8.52	[M − H]^−^	C_34_H_34_O_18_			✓	✓	✓			✓	✓	✓	
Kaempferol 3-[6‴-*p*-coumarylglucosyl-(1-2)-rhamnoside]	740.1951	19.54	[M − H]^−^	C_36_H_36_O_17_			✓	✓	✓		✓	✓	✓	✓	
Coumaroylglucosyl quercitrin	756.1901	17.91	[M − H]^−^	C_36_H_36_O_18_			✓	✓	✓		✓	✓	✓	✓	
Triterpenoid saponin	972.4936	25.74	[M − H]^−^				✓	✓	✓						

a AG – *A. goniocephala*, AB*– A. brachyphylla*, H – *n*-hexane, EA – ethyl acetate, E – ethanol, E_W – ethanol/water, W – water; RT – retention time.

**Table 3:** List of tentatively identified compounds from *Achillea* extracts in the positive ionization mode^a^

Compounds	Mass	RT, min	ESI	Formula	AG_H	AG_EA	AG_E	AG_E_W	AG_W	AB_H	AB_EA	AB_E	AB_E_W	AB_W	Ref.
Pyroglutamic acid	129.0426	1.89	[M + H]^+^	C_5_H_7_NO_3_	✓	✓	✓	✓	✓		✓	✓	✓	✓	
Amino acid 1	129.0791	1.51	[M + H]^+^		✓	✓	✓	✓	✓			✓	✓	✓	
Isoleucine/leucine	131.0944	2.38	[M + H]^+^	C_6_H_12_NO_2_			✓	✓	✓			✓	✓	✓	
Leucine/isoleucin	131.0945	2.57	[M + H]^+^	C_6_H_12_NO_2_			✓	✓	✓			✓	✓	✓	
Coumaric acid	164.0474	7.99	[M + H]^+^	C_9_H_8_O_3_		✓	✓								
Phenylalanine	165.0789	3.95	[M + H]^+^	C_9_H_11_NO_2_			✓	✓	✓			✓	✓	✓	
Tyrosine	181.0738	2.21	[M + H]^+^	C_9_H_11_NO_3_			✓	✓	✓			✓	✓	✓	
Decenoic acid	192.1125	19.32	[M + H]^+^	C_10_H_18_O_2_	✓	✓	✓	✓	✓						
Achillin/matricin	246.1255	24.52	[M + H]^+^	C_15_H_18_O_3_	✓	✓	✓	✓	✓						9
Abscisic aldehyde	248.1413	10.79	[M + H]^+^	C_15_H_20_O_3_	✓	✓	✓	✓	✓						
Dihydroabscisic alcohol	252.1723	25.63	[M + H]^+^	C_15_H_24_O_5_						✓	✓	✓	✓	✓	
Dihydroabscisic acid	266.1516	12.01	[M + H]^+^	C_15_H_20_O_4_	✓	✓	✓	✓	✓						
Naringenin	272.0662	5.53	[M + H]^+^	C_15_H_12_O_5_	✓	✓	✓	✓	✓						
Trihydroxyflavanone	272.0662	8.84	[M + H]^+^	C_15_H_12_O_5_	✓	✓	✓	✓	✓						
Achimilic acid	278.1154	9.43	[M + H]^+^	C_15_H_18_O_5_	✓	✓	✓	✓	✓						
Naringenin dimethyl ether	300.0971	9.70	[M + H]^+^	C_17_H_16_O_5_	✓	✓	✓								
Dihydroxy-dimethoxyflavone	314.0787	25.63	[M + H]^+^	C_17_H_14_O_6_	✓	✓	✓	✓	✓	✓	✓	✓	✓	✓	
Trihydroxy-dimethoxyflavone	330.0739	24.95	[M + H]^+^	C_17_H_14_O_7_	✓	✓	✓	✓	✓	✓	✓	✓		✓	
Eupatorin	344.0896	25.67	[M + H]^+^	C_18_H_16_O_7_	✓	✓	✓	✓	✓	✓	✓	✓	✓	✓	50
Chlorogenic acid	354.0954	8.55	[M + H]^+^	C_16_H_18_O_9_	✓	✓	✓	✓	✓		✓	✓	✓	✓	8, 9 and 29
Quercetin tetramethyl ether	358.1061	26.40	[M + H]^+^	C_19_H_18_O_7_	✓	✓	✓	✓	✓	✓	✓	✓	✓	✓	
*O*-Feruloylquinic acid	368.1107	11.02	[M + H]^+^	C_17_H_20_O_9_	✓	✓	✓	✓	✓		✓	✓	✓	✓	
Dihydroxy-tetramethoxyflavone	374.1	25.90	[M + H]^+^	C_19_H_18_O_8_	✓	✓	✓	✓	✓						
Artemetin	388.117	26.72	[M + H]^+^	C_20_H_20_O_8_	✓	✓	✓	✓	✓						
Vitexin/isovitexin	432.1052	15.51	[M + H]^+^	C_21_H_20_H_10_		✓	✓	✓	✓		✓	✓	✓	✓	29
Apigenin-*O*-pentoside	446.0846	18.16	[M + H]^+^	C_21_H_18_O_11_	✓		✓	✓	✓				✓		
Kaempferol *O*-hexoside	448.1	13.31	[M + H]^+^	C_21_H_20_O_11_	✓	✓	✓	✓	✓		✓	✓	✓	✓	9
Luteolin-*O*-hexoside	448.1004	15.68	[M + H]^+^	C_21_H_20_O_11_	✓	✓	✓	✓	✓		✓	✓	✓	✓	9 and 29
Trihydroxy-methoxyflavone-*O*-glucoside isomer 1	462.1157	16.15	[M + H]^+^	C_22_H_22_O_11_		✓	✓	✓	✓		✓	✓	✓	✓	
Scoparin	462.1163	13.46	[M + H]^+^	C_22_H_22_O_11_	✓	✓	✓	✓	✓						
Dicaffeoyl-quinolactone	498.1167	15.20	[M + H]^+^	C_25_H_22_O_11_	✓	✓	✓	✓	✓		✓	✓	✓	✓	
Dicaffeoylquinic acid	516.1271	15.19	[M + H]^+^	C_25_H_24_O_12_	✓		✓	✓	✓		✓	✓	✓	✓	
Fatty acid 3	526.3269	24.94	[M + H]^+^		✓	✓	✓	✓	✓						
Fatty acid 2	530.3584	24.55	[M + H]^+^		✓	✓	✓	✓	✓						
Fatty acid 1	562.3481	22.14	[M + H]^+^		✓	✓	✓	✓	✓						
*C*-Hexosyl-*C*-pentosyl apigenin 1	564.1474	12.66	[M + H]^+^	C_26_H_28_O_14_	✓	✓	✓	✓	✓		✓	✓	✓	✓	9
Apigenine derivate 2	564.1474	15.02	[M + H]^+^	C_26_H_28_O_14_			✓	✓	✓		✓	✓	✓	✓	
Hydroxyapigenin-xylosyl-galactoside	580.1419	11.81	[M + H]^+^	C_26_H_28_O_15_	✓	✓	✓	✓	✓			✓	✓	✓	
*C*-Hexosyl-*C*-pentosylluteolin	580.1424	11.65	[M + H]^+^	C_26_H_28_O_15_	✓	✓	✓	✓	✓				✓	✓	9
Apigenin di-glucoside 2	594.1581	16.60	[M + H]^+^	C_27_H_30_O_15_			✓	✓	✓		✓	✓	✓	✓	
Vitexin-*O*-hexoside	594.1588	13.92	[M + H]^+^	C_27_H_30_O_15_	✓	✓	✓	✓	✓		✓	✓	✓	✓	
Rutin	610.1532	16.54	[M + H]^+^	C_27_H_30_O_16_			✓	✓	✓			✓	✓		8 and 9
Prenylapigenin 7-[rhamnosyl-(1-6)-glucoside]	646.2232	4.65	[M + H]^+^	C_32_H_38_O_14_		✓	✓	✓	✓						

a AG – *A. goniocephala*, AB *– A. brachyphylla*, H – *n*-hexane, EA – ethyl acetate, E – ethanol, E_W – ethanol/water, W – water; RT – retention time.

The PCA score plots demonstrated pronounced differentiation among the ten species – extract combinations in both ionisation modes (Fig. 1A and 2A). The first two principal components explained 76.2% of the total variance in the negative ionisation mode (PC1 = 51.3%; PC2 = 24.9%) and 83.6% in the positive ionisation mode (PC1 = 64.8%; PC2 = 18.8%). PERMANOVA further demonstrated significant overall differences among the predefined extract groups in both datasets (*p* = 0.001). These results confirm substantial differentiation in the overall metabolite fingerprints of the analysed samples; however, as the reported PERMANOVA represents an overall group comparison, the independent contributions of species, extraction solvent and their interaction cannot be distinguished from these values alone.

**Fig. 1:**
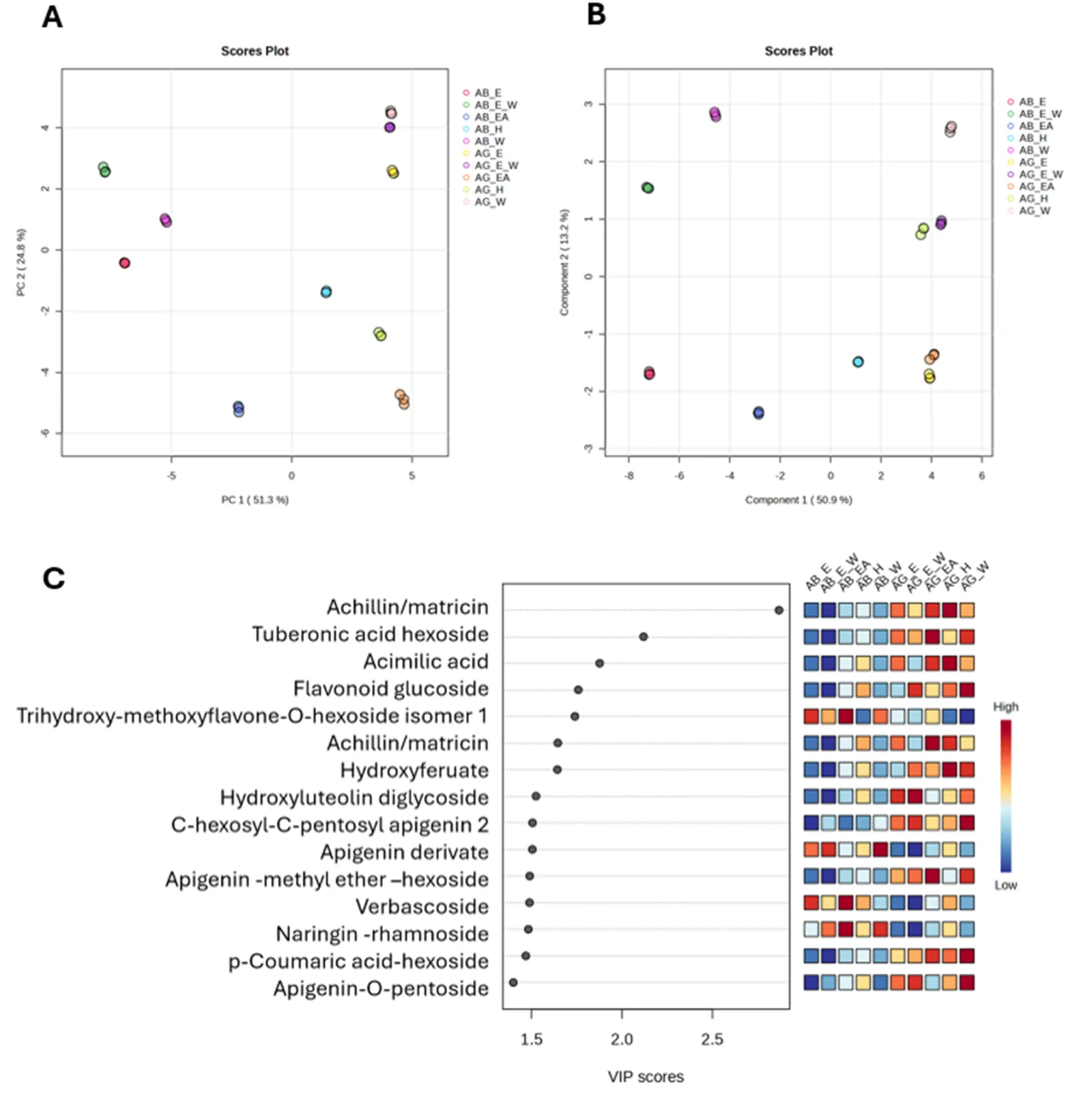
(A) PCA analysis; (B) PLS-DA analysis and (C) 15 features with VIP scores > 1 of the five extracts of *Achillea* species in the negative ionization mode (AG *– A. goniocephala*, AB *– A. brachyphylla*, H – *n*-hexane, EA – ethyl acetate, E – ethanol, E_W – ethanol/water, W – water).

**Fig. 2:**
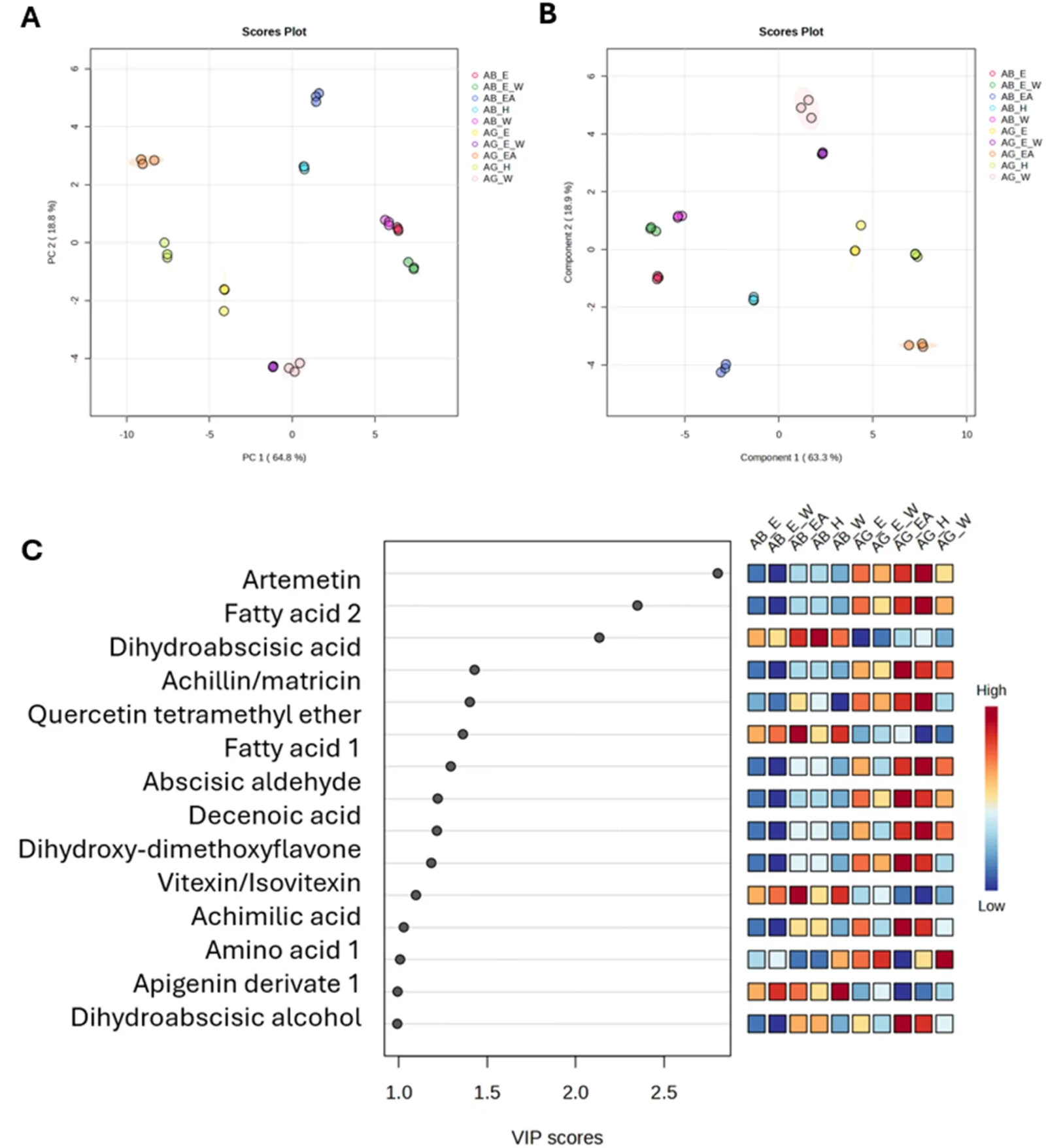
(A) PCA analysis; (B) PLS-DA analysis and (C) 15 features with VIP scores > 1 of the five extracts of *Achillea* species in the positive ionization mode (AG – *A. goniocephala*, AB – *A. brachyphylla*, H – *n*-hexane, EA – ethyl acetate, E – ethanol, E_W – ethanol/water, W – water).

The corresponding PLS-DA models provided a complementary supervised representation of sample separation (Fig. 1B and 2B). VIP analysis identified 15 features with VIP scores >1 in each ionisation mode as the strongest contributors to group discrimination (Fig. 1C and 2C). Notably, achillin/matricin and achimilic acid were among the discriminant features in both datasets, consistent with the contribution of species-associated chemical differences to the overall multivariate structure alongside pronounced extraction-dependent variation.

The heatmaps provided further insights into the metabolite distribution underlying this multivariate differentiation (Fig. 3 and 4). Hierarchical clustering of the metabolites revealed groups of features with distinct abundance patterns across the ten extracts rather than a uniform distribution throughout the dataset. The negative- and positive – ionisation profiles, therefore, indicate that the observed separation reflects a combination of extraction-dependent enrichment and species-associated metabolite patterns. This observation is consistent with the VIP results, and provides a basis for examining the major phytochemical classes and candidate species-discriminating constituents in greater detail.

**Fig. 3:**
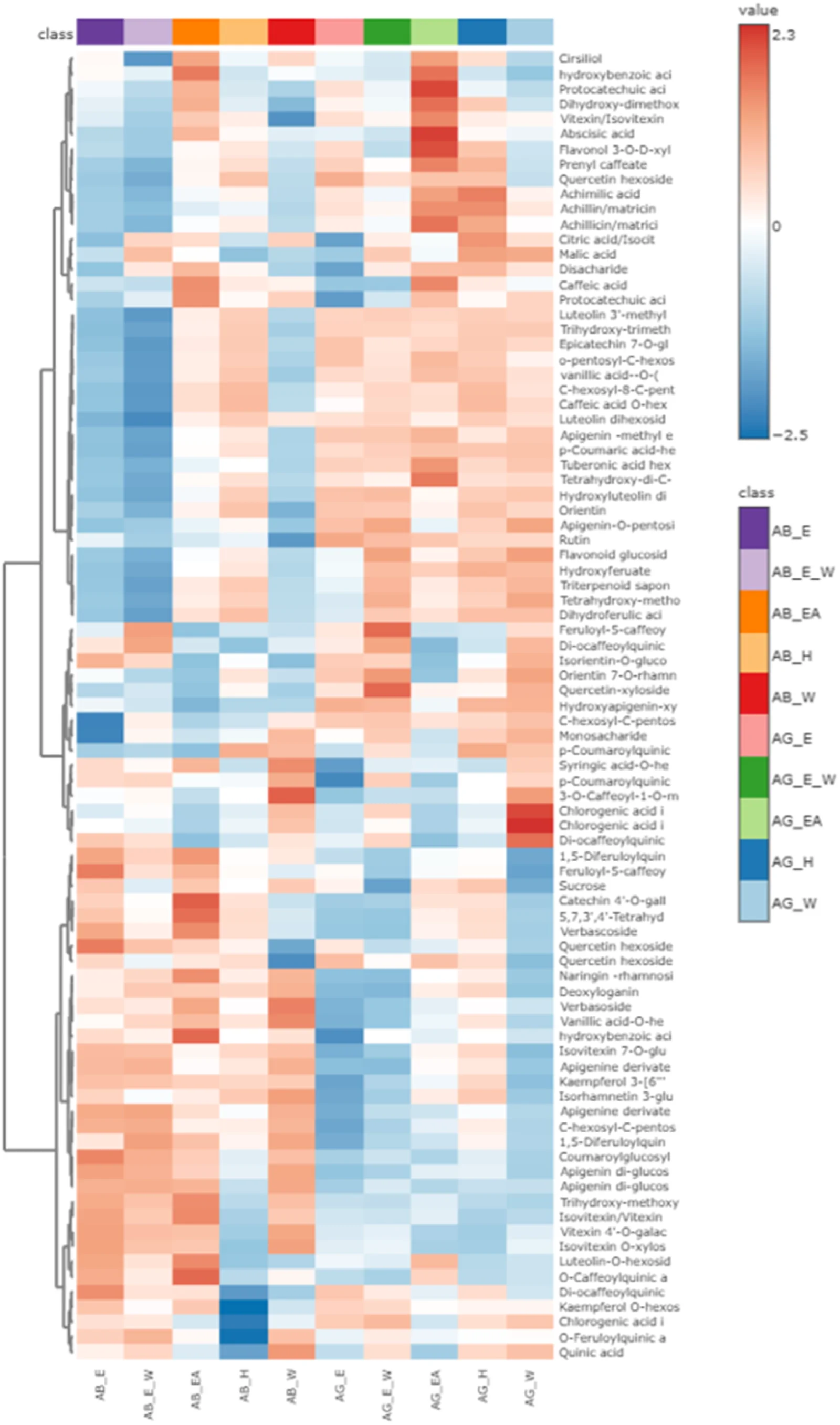
Heat map of the five extracts of *Achillea* species obtained in the negative ionization mode (AG – *A. goniocephala*, AB – *A. brachyphylla*, H – *n*-hexane, EA – ethyl acetate, E – ethanol, E_W – ethanol/water, W – water).

**Fig. 4:**
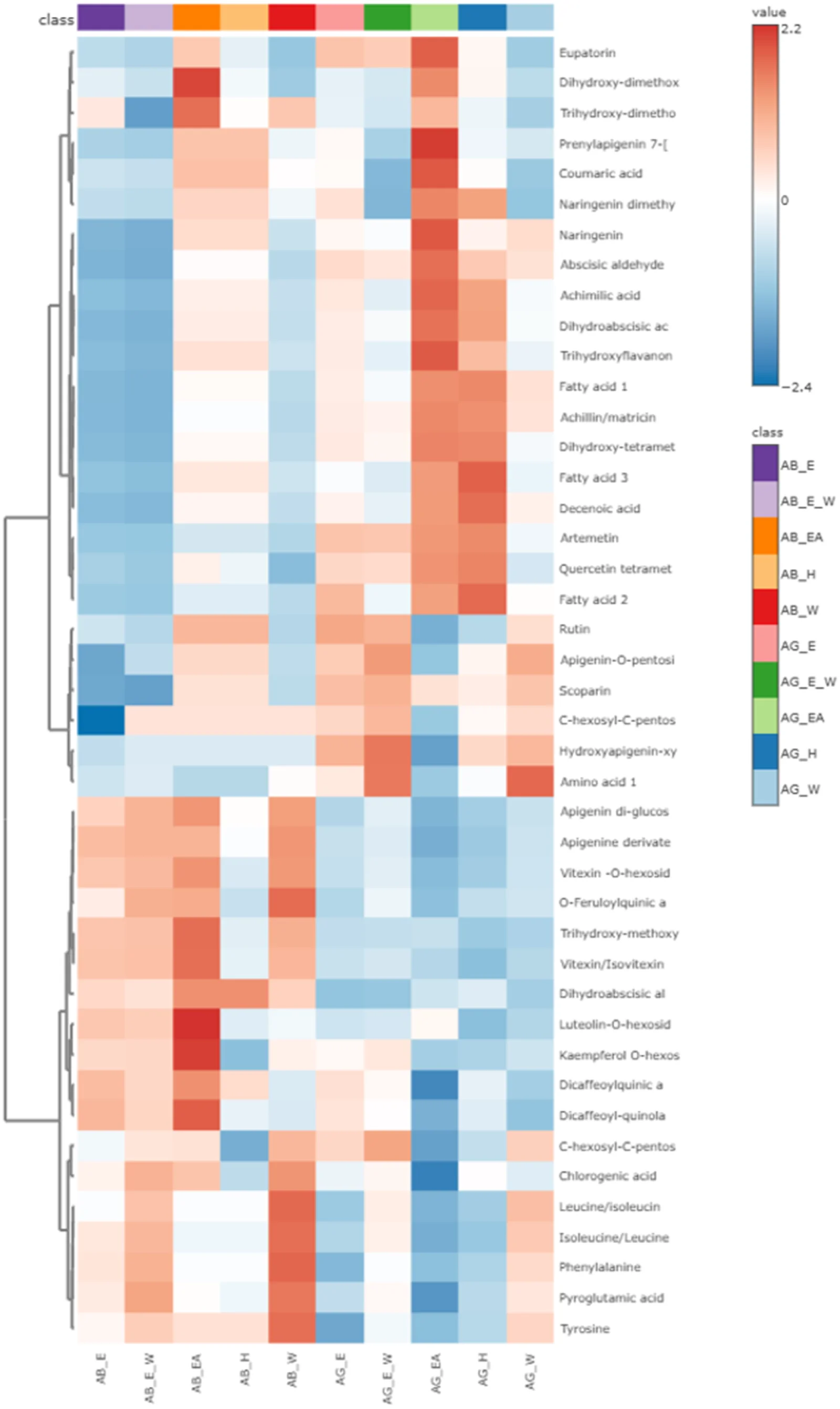
Heat map of the five extracts of *Achillea* species in the positive ionization mode (AG – *A. goniocephala*, *AB* – *A. brachyphylla*, H – *n*-hexane, EA – ethyl acetate, E – ethanol, E_W – ethanol/water, W – water).

Acylquinic acids and related hydroxycinnamic acid derivatives represent one of the most prominent groups within the annotated metabolome. Multiple chlorogenic acid isomers were detected together with caffeoyl-, *p*-coumaroyl- and feruloylquinic derivatives, dicaffeoylquinic acids and mixed feruloyl-caffeoylquinic acid derivatives. Simpler phenolic constituents, including caffeic and protocatechuic acids and their conjugated forms, were also represented in the profiles. The prominence of these compounds is consistent with previous phytochemical investigations of the genus. LC-MS/MS analysis of *A. coarctata* and *A. monocephala* identified chlorogenic acid and related phenolic constituents among the characteristic phytochemicals of the investigated species,^8^ while high-resolution profiling of *A. aleppica* and *A. santolinoides* revealed a particularly diverse array of mono- and di-acylquinic acid derivatives.^9^ A comparative investigation of twelve Anatolian *Achillea* taxa, including *A. goniocephala*, likewise demonstrated the importance of chlorogenic acid and related phenolics in the chemical differentiation of the genus.^12^

Flavonoids constituted another major component of the metabolite profiles and were represented by both glycosylated and aglycone forms. The annotated compounds included *C*- and *O*-glycosylated flavones and flavonols related to vitexin/isovitexin, orientin, luteolin and apigenin, together with rutin and kaempferol- and quercetin-derived glycosides. More lipophilic methoxylated flavones, including cirsiliol-, eupatorin- and artemetin-related constituents, were also detected. This chemical diversity agrees well with previous reports for *Achillea*, in which *C*-glycosylated flavones, *O*-glycosylated flavones and flavonols, and less polar flavonoid aglycones were identified as important components of the genus.^9^ Targeted LC-MS/MS investigations have similarly reported rutin, luteolin, apigenin and related flavonoids in different *Achillea* species.^8^ The present findings, therefore, further support the considerable structural diversity of the flavonoid fraction within the genus.

A particularly notable feature of the present dataset was the occurrence of species-associated qualitative differences between the two taxa. According to the current presence/absence matrices, achillin/matricin and achimilic acid were tentatively annotated across the *A. goniocephala* extracts but were not detected in the corresponding *A. brachyphylla* extracts in either ionisation mode. Achillicin/matricin and prenyl caffeate showed a similarly restricted distribution to *A. goniocephala* in the negative ionisation mode. Conversely, several features were detected exclusively among the *A. brachyphylla* extracts, including deoxyloganin, the phenylethanoid glycoside verbascoside and naringin rhamnoside, although their occurrence varied among extraction solvents; dihydroabscisic alcohol was additionally characteristic of the *A. brachyphylla* profile in the positive ionisation mode. These presence/absence patterns suggest that the two species differ not only in the relative distribution of shared metabolites but also in their qualitative phytochemical signatures. In particular, the occurrence of sesquiterpene-lactone-related constituents in *A. goniocephala* provides a potentially useful chemotaxonomic distinction between the two taxa. Sesquiterpene lactones are well-established constituents of the genus *Achillea*,^9^ supporting the chemical plausibility of this pattern. Nevertheless, because the present assignments remain tentative and non-detection in untargeted LC-MS may also reflect extraction selectivity, ionisation efficiency or detection limits, these compounds should be regarded as candidate species-discriminating markers rather than definitive chemotaxonomic markers until confirmed by targeted analyses and authentic standards.

Clear differences were also apparent among extracts obtained with solvents of contrasting polarity. The ethanol/water and ethanol extracts showed a greater representation of phenolic acids and glycosylated flavonoids, whereas ethyl acetate yielded a more selective profile containing moderately polar constituents. The *n*-hexane fractions were comparatively enriched in lipophilic features, while aqueous extraction favoured highly polar metabolites, including organic acids and sugars. Comparable extraction-dependent differences have been reported in other members of the genus. Distinct metabolite profiles were observed among ethyl acetate, methanol and aqueous extracts of *A. aleppica* and *A. santolinoides*,^9^ while *A. maritima* extracts prepared using *n*-hexane, ethyl acetate, ethanol, ethanol/water and water similarly showed clear differentiation between polar and non-polar phytochemical fractions.^11^ These findings reinforce the importance of examining multiple extraction systems when characterising chemically complex and comparatively understudied plant species.

The LC-MS-QTOF profiles also provide a useful compositional context for the spectrophotometric TPC and TFC results. In *A. goniocephala*, water and ethanol/water extracts showed the highest TPC, whereas the ethanol/water extract exhibited the highest TFC. In *A. brachyphylla*, ethanol was particularly effective for flavonoid recovery. The chromatographic data indicate, however, that these differences reflect not only variations in the overall amount of Folin–Ciocalteu- or flavonoid-reactive material but also changes in the qualitative distribution of individual metabolite classes. In particular, the alcoholic and hydroalcoholic extracts showed broader representation of phenolic acids and flavonoid glycosides, whereas the aqueous extracts also contained numerous other highly polar constituents. The spectrophotometric and chromatographic approaches, therefore, provide complementary information: the TPC and TFC describe aggregate assay responses, while LC-MS-QTOF reveals the underlying compositional diversity of the extracts.

Taken together, the LC-MS-QTOF and multivariate results demonstrate substantial chemical differentiation across the two *Achillea* species and their respective extracts. Acylquinic acid derivatives and flavonoids represented major components of the shared phytochemical profile, whereas their relative distribution, together with that of methoxylated flavones, terpenoid-related compounds and other metabolites, varied considerably among extraction systems. Importantly, the species-associated occurrence of several tentatively annotated constituents further indicates that the observed chemical differentiation cannot be attributed to extraction conditions alone. Thus, the solvent-resolved strategy applied here revealed both extraction-dependent variations and potentially informative qualitative differences between *A. goniocephala* and *A. brachyphylla*, which would be less apparent from the analysis of a single extract. At the same time, because the metabolite assignments are predominantly tentative and the LC-MS signal patterns do not represent absolute concentrations, conclusions regarding individual marker compounds should be considered provisional until confirmed by targeted quantification, diagnostic fragmentation and, where appropriate, comparison with authentic standards.

### Antioxidant activity

The antioxidant properties of *A. brachyphylla* and *A. goniocephala* differed markedly across the species – extract combinations and depended strongly on the assay employed (Table 4). Polar extracts generally exhibited the strongest radical-scavenging and reducing capacities, whereas metal-chelating and phosphomolybdenum activities followed partly different patterns. Thus, no single extract was uniformly superior across all antioxidant assays, emphasising the multidimensional nature of the antioxidant response.

**Table 4:** Antioxidant activity of the tested extracts^a^

Species	Extracts	DPPH^b^ (mg TE per g)	ABTS^b^ (mg TE per g)	CUPRAC^b^ (mg TE per g)	FRAP^b^ (mg TE per g)	MC^c^ (mg EDTAE per g)	PBD^d^ (mmol TE per g)
*A. brachyphylla*	*n*-Hexane	3.51 ± 0.62^f^	62.01 ± 2.54^e^	59.57 ± 1.12^h^	45.36 ± 1.58^g^	15.00 ± 1.34^f^	2.77 ± 0.24^c^
	EA	13.20 ± 0.74^e^	96.44 ± 2.47^d^	76.48 ± 1.79^f^	59.15 ± 1.24^f^	21.71 ± 1.77^d^	3.74 ± 0.16^a^
	Ethanol	57.56 ± 1.45^c^	155.33 ± 0.32^c^	125.26 ± 2.93^d^	108.87 ± 5.31^c^	15.74 ± 1.11^e,f^	3.18 ± 0.05^b^
	Ethanol/water	67.00 ± 0.98^a^	191.50 ± 0.63^a^	108.23 ± 1.14^e^	94.60 ± 0.33^d^	23.04 ± 0.44^d^	3.19 ± 0.14^b^
	Water	58.45 ± 1.01^c^	181.33 ± 1.28^b^	69.31 ± 0.77^f,g^	75.65 ± 2.11^e^	26.04 ± 0.23^c^	1.61 ± 0.04^e^
*A. goniocephala*	*n*-Hexane	4.60 ± 0.82^f^	35.55 ± 4.81^g^	59.97 ± 2.03^h^	31.14 ± 1.63^h^	29.49 ± 0.94^b^	2.66 ± 0.21^c^
	EA	6.17 ± 0.43^f^	50.00 ± 5.52^f^	63.65 ± 3.28^g,h^	37.72 ± 2.52^g,h^	30.02 ± 0.24^b^	3.18 ± 0.19^b^
	Ethanol	51.13 ± 1.80^d^	103.73 ± 2.03^d^	209.08 ± 1.48^c^	125.87 ± 2.38^b^	17.87 ± 0.71^e^	3.31 ± 0.06^b^
	Ethanol/water	65.06 ± 0.15^a,b^	157.17 ± 2.42^c^	281.37 ± 5.04^a^	172.43 ± 5.59^a^	31.92 ± 0.28^b^	2.25 ± 0.01^d^
	Water	62.47 ± 0.18^b^	160.53 ± 0.20^c^	235.42 ± 3.24^b^	182.74 ± 7.59^a^	34.95 ± 0.38^a^	1.61 ± 0.07^e^

a values are presented as mean ± SD of three measurements. Means within each column with different letters (a–h) differ significantly (*p* ≤ 0.05).

b mg Trolox equivalents per g of extract.

c mg EDTAE per g of extract.

d mmol Trolox equivalents per g of extract.

Radical scavenging activity was most pronounced in the polar extracts. In the DPPH assay, the ethanol/water extract of *A. brachyphylla* showed the highest activity (67.00 mg TE per g), but did not differ significantly from the corresponding *A. goniocephala* extract (65.06 mg TE per g; *p* > 0.05). Within *A. goniocephala*, the ethanol/water and water extracts were similarly statistically comparable. A clearer differentiation was observed in the ABTS assay, where the *A. brachyphylla* ethanol/water extract exhibited the highest activity (191.50 mg TE per g) and was significantly more active than all other extracts. In *A. goniocephala*, the ethanol/water and water extracts showed comparable ABTS activities, whereas the ethanol extract was significantly less active than both. These results indicate that hydroalcoholic and aqueous extraction generally favoured radical-scavenging activity, although the magnitude of the response differed between the two species. A marked antioxidant potential of hydroalcoholic *A. santolina* extracts has similarly been demonstrated using DPPH and several complementary *in vitro* systems.^28^ In addition, Taşkın *et al.*^29^ showed pronounced extraction-dependent variation in the antioxidant properties of 15 differently prepared extracts of *A. nobilis* subsp. *neilreichii*, demonstrating that both extraction method and solvent selection can substantially affect the resulting biological activity.

A comparable solvent-dependent pattern has recently been reported for *A. maritima*, for which the same sequence of *n*-hexane, ethyl acetate, ethanol, ethanol/water and water extracts was investigated.^11^ In that study, water and ethanol/water extracts showed the strongest DPPH and ABTS activities, whereas *n*-hexane was the least active fraction. However, the results were expressed as IC_50_ values rather than Trolox equivalents, and direct numerical comparison with the present data is therefore not appropriate; the agreement is instead evident at the level of solvent-dependent activity patterns.

The reducing-power assays revealed an even more pronounced distinction between the two species. In CUPRAC, the *A. goniocephala* ethanol/water extract displayed the highest activity (281.37 mg TE per g), significantly exceeding that of the corresponding water and ethanol extracts. Moreover, these three polar *A. goniocephala* extracts showed substantially greater reducing capacities than those of their respective *A. brachyphylla* counterparts. FRAP produced a related pattern: the water and ethanol/water extracts of *A. goniocephala* showed the highest activities and were not significantly different from each other, while ethanol exhibited a significantly lower response. In *A. brachyphylla*, ethanol was the most active extract in both CUPRAC and FRAP. Considerable interspecific variability in antioxidant performance has also been documented among endemic *Achillea* taxa by Gharibi *et al.*,^30^ while Trumbeckaite *et al.*^31^ demonstrated that the antiradical activity of *A. millefolium* was closely associated with active phenolic constituents identified by chromatographic analysis.

The magnitude of the reducing response observed for *A. goniocephala* is also noteworthy in comparison with studies employing the same equivalent-based methodology. Gevrenova *et al.*^9^ reported maximum CUPRAC and FRAP activities of 183.55 and 129.92 mg TE per g, respectively, for the methanolic root extract of *A. santolinoides*, whereas different solvent and plant – part combinations produced substantially lower activities. Although differences in species, plant material, and extraction procedures preclude strict quantitative comparison, these findings reinforce the considerable variability in reducing capacity within the genus and the strong dependence of antioxidant performance on phytochemical composition.

The compositional results provide a useful context for these trends. In *A. goniocephala*, the strongest CUPRAC and FRAP activities occurred in the polar extracts that also exhibited the highest TPC values, while LC-MS-QTOF profiling revealed extensive representation of acylquinic acids, hydroxycinnamic acid derivatives and flavonoid glycosides in these fractions. In *A. brachyphylla*, ethanol combined the highest TFC with the strongest CUPRAC and FRAP responses within the species. These parallel trends are consistent with the contribution of phenolic and flavonoid constituents to reducing capacity. Nevertheless, the relationship was not uniform across all assays. For example, the aqueous *A. brachyphylla* extract exhibited high ABTS activity despite its very low TFC. This lack of a simple correspondence between aggregate phenolic indices and antioxidant response is consistent with the observations for *A. millefolium*, where heterogeneous antioxidant effects and relatively weak correlations with the overall phenolic content suggested an important role for the identity and relative abundance of individual constituents rather than total phenolics alone.^4^

Metal-chelating activity showed a distinctly different distribution. The *A. goniocephala* water extract displayed the highest chelating capacity (34.95 mg EDTAE per g) and was significantly more active than all other extracts. Conversely, its *n*-hexane, ethyl acetate and ethanol/water extracts were statistically comparable despite displaying very different responses in the radical-scavenging and reducing-power assays. Water was also the most effective chelating extract within *A. brachyphylla*. These findings indicate that metal chelation did not simply parallel TPC, TFC or reducing capacity and probably reflects a different combination of extract constituents. Similar assay-dependent behaviour was observed by Gevrenova *et al.*,^9^ who found particularly strong chelating activities in aqueous and ethyl acetate fractions of *A. aleppica* and *A. santolinoides*, including values of 25.37 and 27.37 mg EDTAE per g, respectively.

The phosphomolybdenum assay further demonstrated the absence of a universal solvent ranking. The *A. brachyphylla* ethyl acetate extract showed the highest response (3.74 mmol TE per g) and was significantly more active than the remaining extracts, whereas its ethanol and ethanol/water extracts were statistically comparable. In contrast, the water extracts of both species displayed the lowest phosphomolybdenum values and did not differ significantly from each other. This behaviour contrasts with the strong performance of aqueous and hydroalcoholic extracts in several radical-scavenging and reducing assays. A comparable preference for ethyl acetate was reported for the phosphomolybdenum activity of the aerial parts of both *A. aleppica* and *A. santolinoides*.^9^ Similarly, *A. maritima* showed a different solvent ranking in the phosphomolybdenum assay compared with most of its other antioxidant tests.^11^

Taken together, the six assays demonstrate that the antioxidant properties of the two *Achillea* species cannot be adequately represented by a single assay or predicted solely from TPC or TFC. Polar extraction generally favoured DPPH and ABTS radical scavenging and, particularly in *A. goniocephala*, CUPRAC and FRAP reducing capacity, whereas metal-chelating and phosphomolybdenum activities followed partly independent patterns. This assay-dependent behaviour is consistent with the chemically heterogeneous LC-MS-QTOF profiles and indicates that different groups of constituents may contribute to different components of the overall antioxidant response. Previous work has already demonstrated the antioxidant activity of *A. goniocephala* fractions using DPPH, CUPRAC and FRAP assays,^13^ although the different extraction and fractionation strategy precludes direct quantitative comparison. The present solvent-resolved analysis therefore extends the existing evidence by characterising antioxidant behaviour across a broad polarity range and, importantly, by enabling direct comparison of *A. goniocephala* and the much less investigated *A. brachyphylla* within the same experimental framework.

### Enzyme inhibitory activity

The enzyme inhibitory profiles of *A. brachyphylla* and *A. goniocephala* were strongly dependent on both the target enzyme and extraction solvent (Table 5). Importantly, the solvent yielding the strongest inhibition differed among enzymes, and extracts displaying pronounced activity against one target were not necessarily the most effective against another. This pattern was particularly evident when the cholinesterase, tyrosinase and carbohydrate-hydrolysing enzyme assays were considered together, indicating that enzyme inhibition cannot be predicted simply from extract polarity, TPC or TFC.

**Table 5:** Enzyme inhibitory activity of the tested extracts^a^

Species	Extracts	AChE^b^ (mg GALAE per g)	BChE^b^ (mg GALAE per g)	Tyrosinase^c^ (mg KAE per g)	α-Amylase^d^ (mmol ACAE per g)	α-Glucosidase^d^ (mmol ACAE per g)
*A. brachyphylla*	*n*-Hexane	1.81 ± 0.29^c,d^	na	31.19 ± 2.55^d,e^	0.94 ± 0.06^a^	na
	EA	2.01 ± 0.22^b,c^	4.00 ± 0.07^a^	39.73 ± 0.73^b^	0.87 ± 0.02^a^	0.06 ± 0.01^d^
	Ethanol	2.55 ± 0.16^a^	1.66 ± 0.19^c^	51.51 ± 0.58^a^	0.70 ± 0.05^b^	1.66 ± 0.10^a,b^
	Ethanol/water	1.93 ± 0.01^c^	0.85 ± 0.15^d^	51.14 ± 0.41^a^	0.57 ± 0.01^c^	1.55 ± 0.20^b^
	Water	na^e^	na	37.89 ± 1.07^b,c^	0.09 ± 0.01^e^	0.53 ± 0.01^c^
*A. goniocephala*	*n*-Hexane	1.97 ± 0.06^b,c^	1.87 ± 0.06^c^	32.18 ± 3.36^c,d,e^	0.72 ± 0.01^b^	na
	EA	2.53 ± 0.07^a^	1.63 ± 0.10^c^	27.17 ± 4.47^e^	0.62 ± 0.02^c^	1.74 ± 0.12^a,b^
	Ethanol	2.33 ± 0.04^a,b^	2.62 ± 0.09^b^	49.29 ± 0.40^a^	0.70 ± 0.01^b^	na
	Ethanol/water	2.04 ± 0.01^b,c^	0.88 ± 0.03^d^	50.97 ± 0.62^a^	0.48 ± 0.01^d^	1.93 ± 0.06^a^
	Water	1.44 ± 0.06^d^	na	33.99 ± 0.11^b,c,d^	0.11 ± 0.01^e^	1.86 ± 0.24^a,b^

a values are presented as mean ± SD of three measurements. Means within each column with different letters (a–e) differ significantly (*p* ≤ 0.05).

b mg galantamine equivalents per g of extract.

c mg kojic acid per g of extract.

d mmol acarbose equivalents per g of extract.

e na – not active.

Cholinesterase inhibition showed distinct patterns for AChE and BChE. The highest AChE activities were observed for the ethanol extract of *A. brachyphylla* and the ethyl acetate extract of *A. goniocephala* (2.55 and 2.53 mg GALAE per g, respectively), which did not differ significantly from one another. The ethanol extract of *A. goniocephala* was also statistically comparable with this group exhibiting the highest activity. By contrast, the aqueous *A. brachyphylla* extract showed no detectable AChE inhibition under the assay conditions, while the aqueous *A. goniocephala* extract showed the lowest numerical activity among the active extracts. BChE displayed an even more selective response: the *A. brachyphylla* ethyl acetate extract was significantly more active than all other active extracts (4.00 mg GALAE per g), followed by the *A. goniocephala* ethanol extract. No detectable BChE activity was observed for the water extracts of either species or for the *A. brachyphylla n*-hexane extract.

Such divergence between AChE and BChE inhibition has precedent within the genus. Sevindik *et al.*^32^ demonstrated that fractions of different polarities from *A. millefolium* differed markedly in their cholinesterase effects; notably, the *n*-butanol fraction showed the strongest AChE inhibition, and the bioactivity-guided work identified 6-hydroxyluteolin 7-*O*-glucoside as a potent inhibitor of both AChE and BChE. The endemic *A. cucullata* has also shown pronounced inhibition of both cholinesterases, although its activities were reported as IC_50_ values, and therefore, cannot be compared numerically with the GALAE-based results of the present study.^33^ Within an equivalent-based framework, *A. aleppica* and *A. santolinoides* extracts displayed AChE activities of approximately 0.5–2.8 mg GALAE per g and substantially more variable BChE responses, with ethyl acetate fractions reaching 6.76 mg GALAE per g.^9^ Taken together, these comparisons indicate that cholinesterase inhibition is highly sensitive to both taxonomic identity and extract composition rather than following a uniform solvent-polarity trend.

Tyrosinase inhibition showed markedly different and more consistent solvent dependence. The ethanol and ethanol/water extracts of both species formed a group exhibiting the statistically highest activity, with values ranging from 49.29 to 51.51 mg KAE per g and no significant differences among them. In contrast, the ethyl acetate extract of *A. goniocephala* displayed the lowest activity (27.17 mg KAE per g), while the *A. brachyphylla n*-hexane extract showed an activity of 31.19 mg KAE per g. Thus, unlike the cholinesterases, tyrosinase inhibition was preferentially associated with the alcoholic and hydroalcoholic fractions in both taxa. The same tendency was reported for *A. maritima*, where ethanol was the most active extract against tyrosinase (55.59 mg KAE per g).^11^ In *A. aleppica* and *A. santolinoides*, methanolic and ethyl acetate extracts also showed substantial tyrosinase inhibition, reaching approximately 69–73 mg KAE per g in the most active fractions.^9^ Furthermore, ethanol extracts of the flowers and leaves of *A. filipendulina* have been reported to exhibit appreciable inhibition of tyrosinase together with activity against cholinesterases and carbohydrate-hydrolysing enzymes.^34^ These reports support the broad occurrence of anti-tyrosinase constituents within *Achillea*, while also illustrating substantial species- and extraction-dependent variation.

The α-amylase assay produced a solvent pattern almost opposite to that observed for several antioxidant assays. The *n*-hexane and ethyl acetate extracts of *A. brachyphylla* showed the strongest inhibition (0.94 and 0.87 mmol ACAE per g, respectively) and were statistically indistinguishable. The *n*-hexane extract of *A. goniocephala* and the ethanol extracts of both species formed a lower but statistically comparable group, whereas the water extracts showed only minimal α-amylase inhibition and did not differ significantly from one another. This preference for non-polar to moderately polar fractions suggests that the constituents contributing to α-amylase inhibition differ from those primarily associated with the strong radical-scavenging and reducing activities of the polar extracts. A related pattern was reported for *A. maritima*, in which ethyl acetate was the most active α-amylase inhibitor (0.66 mmol ACAE per g).^11^ By comparison, solvent extracts of *A. aleppica* and *A. santolinoides* tested using the same equivalent-based assay generally showed α-amylase activities ≤0.40 mmol ACAE per g.^9^ Although differences in plant material and experimental conditions preclude strict cross-study ranking, the relatively high α-amylase inhibition of the *A. brachyphylla n*-hexane and ethyl acetate fractions is therefore noteworthy.

The α-glucosidase results differed again from the α-amylase pattern. The highest numerical activity was obtained for the *A. goniocephala* ethanol/water extract (1.93 mmol ACAE per g); however, it was not significantly different from the *A. goniocephala* water and ethyl acetate extracts or the *A. brachyphylla* ethanol extract. The *A. brachyphylla* ethanol/water extract exhibited an intermediate activity, whereas its aqueous and particularly ethyl acetate extracts were significantly weaker. No detectable α-glucosidase inhibition was observed either for the *n*-hexane extract or for the ethanol extract of *A. goniocephala*. This heterogeneous distribution is consistent with previous evidence that α-glucosidase inhibition occurs in several *Achillea* taxa but can be highly dependent on both species and phytochemical composition. An aqueous-ethanolic extract of the endemic *A. cucullata*, for example, inhibited α-glucosidase with an IC_50_ of 24.75 µg ml^−1^,^33^ while a hydroalcoholic *A. millefolium* extract produced 55% α-glucosidase inhibition at 1 mg mL^−1^ in a study combining *in vitro* and *in vivo* antidiabetic approaches.^35^ Because these studies used different response metrics, they provide evidence for the occurrence of activity but do not allow direct quantitative comparison with the ACAE values reported here.

Bioactivity-guided studies further indicate that flavonoids may represent one of several chemical groups capable of contributing to α-glucosidase inhibition within the genus. Ezzat and Salama^36^ isolated several flavonoid-related compounds, including isovitexin and an acacetin *C*-glycoside, from an ethanolic extract of *A. fragrantissima*; the newly characterised *C*-glycosylated flavonoid showed particularly strong α-glucosidase inhibition in their assay. This observation is relevant to the present LC-MS-QTOF dataset, which also contained numerous flavone *C*- and *O*-glycosides, including vitexin/isovitexin-related, apigenin- and luteolin-derived constituents. Nevertheless, the current LC-MS data are qualitative/semi-quantitative and the extracts contain complex mixtures; consequently, the enzyme inhibitory effects cannot be assigned to individual flavonoids without targeted isolation, quantification and activity-guided testing.

More broadly, the enzyme results show only partial correspondence with the TPC, TFC and antioxidant data. This is particularly clear for *A. brachyphylla*: its *n*-hexane extract had low TPC and TFC but produced the strongest α-amylase response, whereas its ethyl acetate extract was the strongest BChE inhibitor. Conversely, the highly phenolic aqueous *A. goniocephala* extract was among the strongest α-glucosidase inhibitors but displayed no detectable BChE activity and only weak α-amylase inhibition. The ethanol and ethanol/water extracts, meanwhile, were consistently among the strongest tyrosinase inhibitors. These contrasting patterns indicate that aggregate phenolic abundance alone does not account for enzyme inhibition, and they are consistent with the solvent-resolved LC-MS profiles, which showed substantial shifts in the relative representation of phenolic acids, flavonoids, terpenoid-related metabolites and other constituents across the extraction systems.

Taken together, the enzyme inhibitory results demonstrate pronounced target-specific differences associated with both species identity and extraction solvent. Neither *A. brachyphylla* nor *A. goniocephala* showed a universally superior enzyme inhibitory profile: *A. brachyphylla* was particularly notable for BChE and α-amylase inhibition in the ethyl acetate and *n*-hexane fractions, whereas *A. goniocephala* showed comparatively strong α-glucosidase activity in several medium-to-polar extracts. Tyrosinase inhibition represented the clearest common pattern between the species, with ethanol and ethanol/water extracts forming the highest statistical group in both taxa. These findings extend previous evidence for multi-target enzyme inhibition within *Achillea*^32–36^ and, more importantly, demonstrate that the biological profiles of these two comparatively understudied species change substantially across the extraction systems. As the present assays are *in vitro* and expressed relative to reference inhibitors, the results should be interpreted as comparative measures of enzyme inhibitory capacity rather than as evidence of therapeutic efficacy.

### Molecular docking analysis

Molecular docking identified several promising interactions across the five enzyme targets, with binding scores ranging from −9.9 to −3.9 kcal mol^−1^ (Table S2). Because docking scores provide relative estimates rather than experimentally validated binding free energies, the results are interpreted primarily in terms of relative binding and binding-mode consistency, followed by MD simulations of the top-ranked complexes.^37^

For BChE, orientin was the strongest candidate, exhibiting the most favorable docking score (−9.9 kcal mol^−1^) and a low RMSD of 0.41 Å. Multiple hydrogen bonds with Asp70, Trp82, Thr120, Trp430, and His438, along with π-stacking with Phe329 (Fig. 5A), supported its predicted binding mode. These interactions involve residues distributed within the BChE gorge,^38^ and are consistent with the ability of aromatic and polyhydroxylated ligands to establish complementary polar and hydrophobic contacts within the enzyme active-site gorge.^39^ The structurally related 3,5-diferuloylquinic acid also showed strong binding (−9.6 kcal mol^−1^) (Fig. 5B). Both 3,5-diferuloylquinic acid and orientin were selected for MD simulations because of their greater docking score and well-defined pose.

**Fig. 5:**
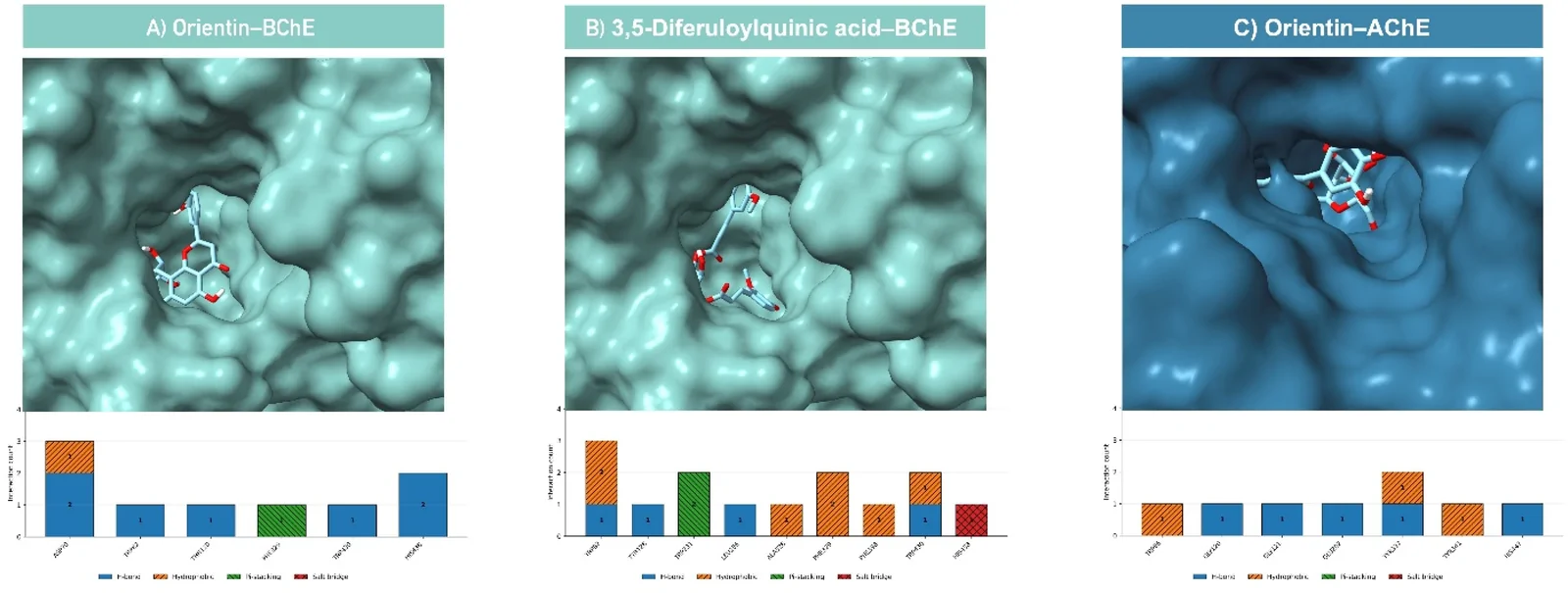
Molecular docking poses and predicted protein–ligand interactions of A. orientin–BChE, B. 3,5-diferuloylquinic acid–BChE, and C. orientin–AChE complexes. Surface representations show ligand binding within the active-site cavities, while the corresponding interaction diagrams summarize hydrogen bond, hydrophobic, π-stacking, and salt – bridge interactions.

Similarly, orientin emerged as the top ligand against AChE, with a docking score of −9.4 kcal mol^−1^ and an RMSD of 0.98 Å. The ligand formed hydrogen bonds with Gly120, Gly121, Glu202, Tyr337, and His447 and hydrophobic contacts with Trp86, Tyr337, and Tyr341 (Fig. 5C). These residues encompass functionally important regions of the AChE catalytic gorge, including Glu202 and His447, while aromatic contacts with Trp86 and Tyr337 may contribute to stabilization within the gorge.^40^ Chlorogenic acid was the second-ranked ligand (−8.8 kcal mol^−1^), but its weaker score supports the selection of orientin as the representative AChE complex for subsequent MD simulations.

For α-amylase, 3,5-diferuloylquinic acid produced the strongest docking score (−9.2 kcal mol^−1^), forming hydrogen bonds with Gln63, Thr163, His201, Asp300, and His305, together with extensive hydrophobic contacts involving Trp59 and salt – bridge interactions involving His299/His305. The combination of polar interactions and aromatic/hydrophobic contacts suggests favorable occupation of the substrate-binding region.^41^ Verbascoside (−8.6 kcal mol^−1^) and cirsiliol (−8.4 kcal mol^−1^) also showed favorable scores but were inferior to 3,5-diferuloylquinic acid. Accordingly, the 3,5-diferuloylquinic acid-α-amylase complex was selected for MD simulation.

For α-glucosidase, orientin was the best-ranked compound, with a docking score of −8.8 kcal mol^−1^ and an exceptionally low RMSD of 0.05 Å. It established a dense hydrogen bond network involving Leu252, Gly253, Gly254, Met289, Tyr291, Arg334, and Arg352, complemented by hydrophobic interactions with Phe350 and a π-cation interaction with Arg352. The combination of numerous polar contacts and aromatic stabilization provides a plausible structural basis for its favorable docking score.^42^

Lastly, the tyrosinase target displayed generally weaker docking scores than those of the other enzymes. Nevertheless, 3,5-diferuloylquinic acid was the strongest candidate (−6.2 kcal mol^−1^), followed by verbascoside (−6.1 kcal mol^−1^) and chlorogenic acid (−5.9 kcal mol^−1^). The predicted 3,5-diferuloylquinic acid complex involved hydrogen bonding with Asn57 and Val218, hydrophobic contacts with His208, Val218, and Ala221, π-stacking with His208, and an additional interaction involving Arg209. These interactions place the ligand within the structurally characterized tyrosinase active-site region. The relatively weak docking score compared with the cholinesterase and carbohydrate-hydrolyzing enzymes suggests that the predicted interaction is less favorable.

### Molecular dynamics simulation analysis

The 100 ns molecular dynamics simulations revealed distinct dynamic behaviors among the five complexes (Fig. 6A). RMSD analysis indicated that C2 and C3 maintained the most conserved global conformations, with mean backbone RMSD values of 0.32 and 0.29 Å, respectively, whereas C1 showed a gradual increase to an average of 0.42 Å. In contrast, C4 exhibited a marked conformational transition during the final 20 ns, reflected by an increase in RMSD to 1.07 Å, while C5 showed a progressive increase to 0.65 Å, suggesting greater structural adaptation during the simulation. Such RMSD changes are generally interpreted as conformational rearrangements rather than as direct evidence of complex instability.^43^

**Fig. 6:**
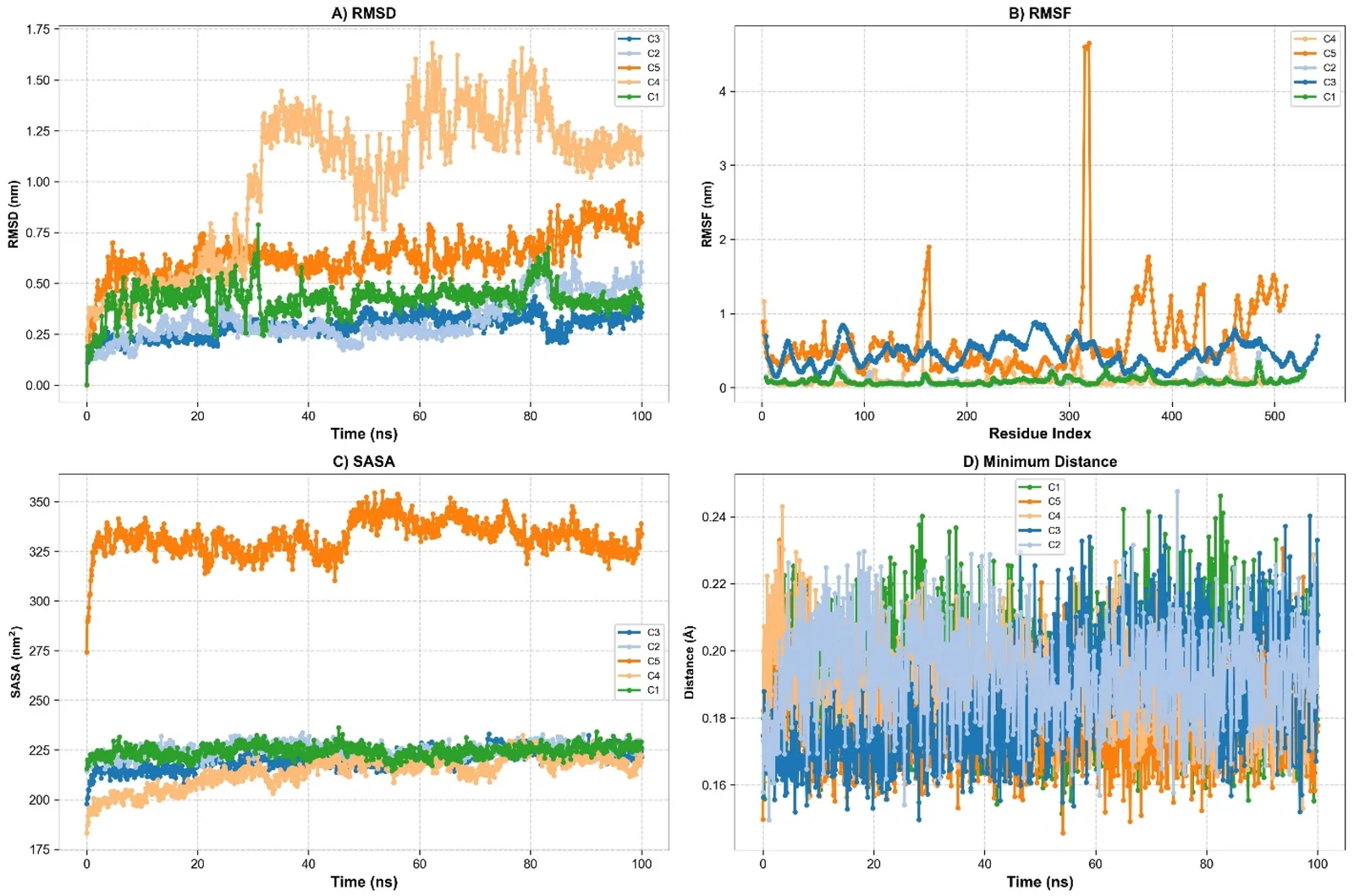
Molecular dynamics simulation profiles of the protein–ligand complexes over 100 ns, showing (A) RMSD, (B) RMSF, (C) solvent-accessible surface area (SASA), and (D) minimum distance. The trajectories indicate generally stable complexes, with C4 exhibiting greater global structural fluctuations and C5 showing pronounced residue-level flexibility. C1: orientin–BChE; C2: 3,5-diferuloylquinic acid–BChE; C3: orientin–AChE; C4: 3,5-diferuloylquinic acid–amylase; and C5: orientin–glucosidase.

RMSF analysis revealed that C1 and C2 displayed low residue-level fluctuations, indicating a relatively rigid protein framework. C3 showed moderate flexibility localized around residues 265–271, whereas C5 exhibited pronounced mobility at residues 315–319, suggesting enhanced local flexibility in this region. Interestingly, despite its higher RMSD, C4 retained low overall RMSF values (Fig. 6B), implying that its late structural change likely involved a collective conformational shift rather than widespread local disorder, consistent with the complementary interpretation of RMSD and RMSF metrics in MD simulations.^44^

The SASA profiles were largely stable for C1 and C2, indicating preserved solvent exposure throughout the simulation. C3 showed a modest increase in SASA during the final 20 ns, coinciding with its late conformational adjustment. More pronounced increases were observed for C4 and C5 (Fig. 6C), suggesting greater solvent exposure associated with their structural rearrangements. SASA variations were used to monitor changes in protein solvent exposure during the simulations.

The minimum distance analysis shows that the ligands remain in close proximity to their respective protein binding sites throughout the 100 ns simulations, with distances generally fluctuating around 0.16–0.21 Å and occasional excursions toward 0.24–0.25 Å (Fig. 6D). The absence of a sustained increase in distance suggests that none of the ligands exhibits clear separation from the binding region during the simulation. Thus, the relatively persistent ligand–binding site proximity provides additional evidence for the maintenance of the docked complexes and their structural stability.

Hydrogen-bond analysis demonstrated that all complexes possessed dynamic interaction networks. C3 maintained the highest average number of hydrogen bonds during most of the trajectory before a late decrease, whereas C1 and C2 showed lower but persistent interactions. C4 and C5 exhibited substantial temporal fluctuations (Fig. 7), consistent with continuous reorganization of ligand–protein contacts during conformational adaptation. Because hydrogen bonds are dynamic in nature, fluctuations in their number were interpreted as continuous interaction remodeling rather than, on their own, as evidence of binding instability.^45^

**Fig. 7:**
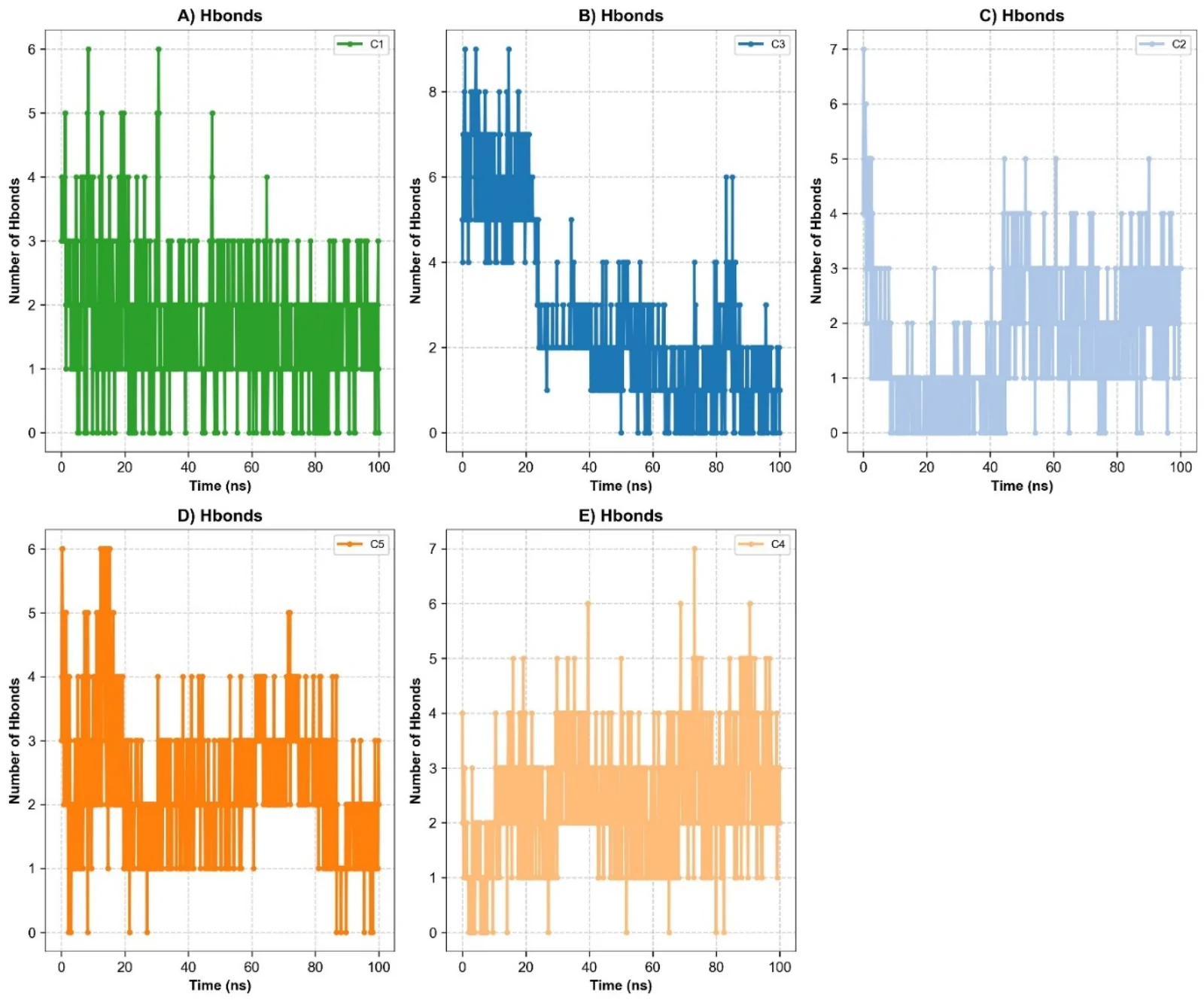
Time evolution of the number of hydrogen bonds (H-bonds) observed for the five systems (C1–C5) over a 100 ns molecular dynamics simulation. Panels (A–E) correspond to C1, C3, C2, C5, and C4, respectively. C1: orientin–BChE; C2: 3,5-diferuloylquinic acid–BChE; C3: orientin–AChE; C4: 3,5-diferuloylquinic acid–amylase; and C5: orientin–glucosidase.

Thus, C2 and C3 displayed the most favorable dynamic profiles, combining low global conformational deviations with limited residue-level flexibility. C1 remained structurally conserved despite gradual adaptation, whereas C4 underwent a pronounced late conformational transition and C5 showed the greatest localized flexibility. Collectively, these results indicate differential conformational responses among the complexes, with C2 and C3 exhibiting the highest degree of structural conservation over the 100 ns simulation.

### Principal component analysis

PCA of the Cα trajectories revealed clear differences in the extent of collective conformational sampling among the five complexes. PCA captures the dominant collective motions contributing to the overall conformational fluctuations, and is therefore complementary to RMSD and RMSF analyses.^46,47^

The C1 complex exhibited the most restricted conformational sampling, with a combined PC1–PC2 dispersion of 1.277 nm, followed by C2 (1.638 nm) and C3 (1.936 nm). C4 showed broader sampling (2.953 nm), whereas C5 displayed a markedly expanded conformational distribution (13.727 nm). Thus, the PCA results indicate progressively greater collective conformational flexibility from C1/C2 to C4, with C5 exhibiting substantially greater conformational heterogeneity. However, these differences in PCA amplitude should be interpreted as differences in sampled conformational space rather than as direct measures of binding stability or affinity.^47^

The PC1–PC2 projections of C1–C3 are consistent with relatively constrained conformational ensembles, whereas the broader distributions observed for C4 and, particularly, C5 suggest sampling of more diverse collective states (Fig. 8). The near-zero PC1–PC2 correlations in all systems indicate that the two principal modes are essentially orthogonal and describe largely independent components of the observed motion.^47^

**Fig. 8:**
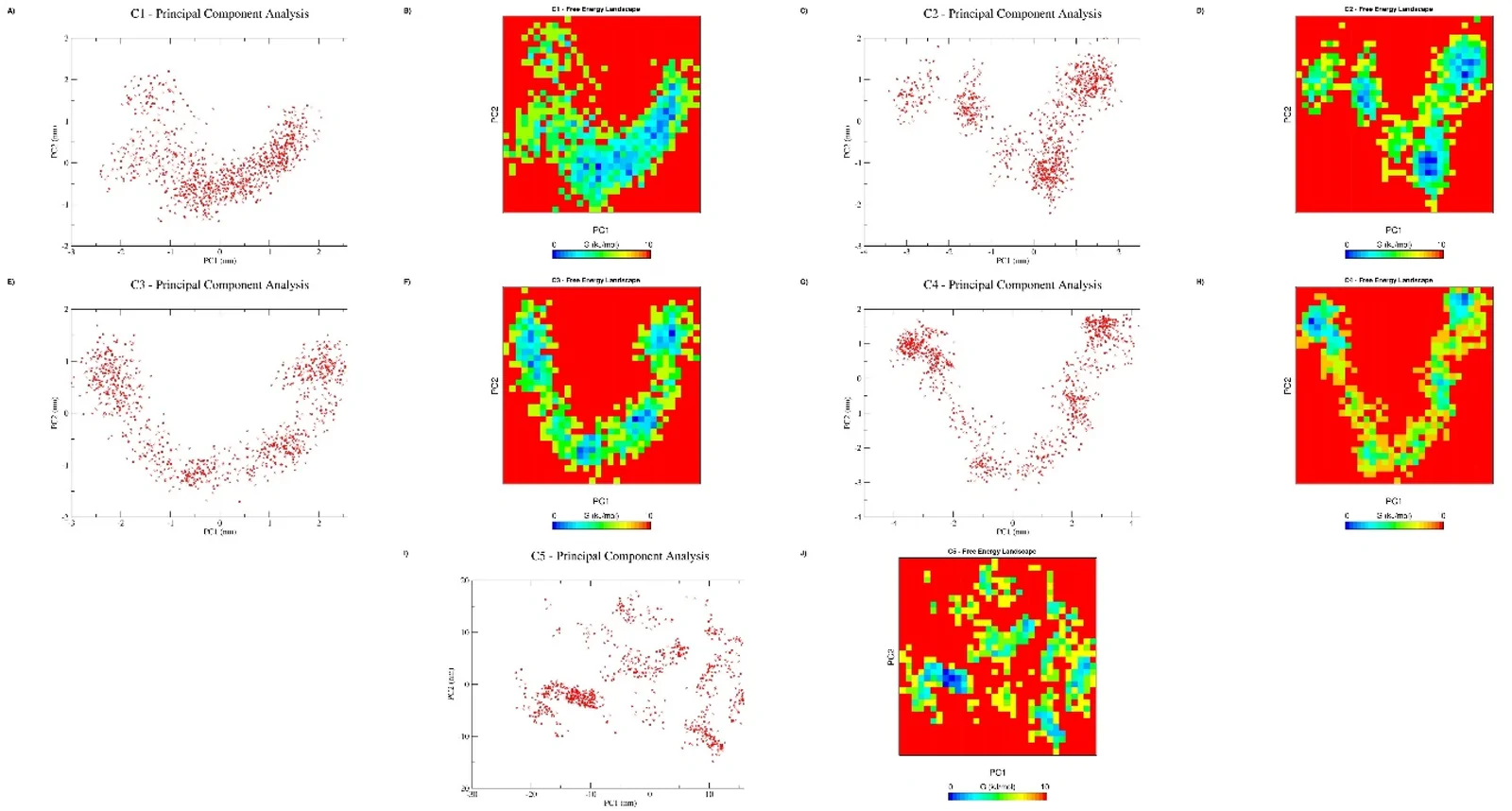
Principal component analysis (PCA) score plots and corresponding PC1–PC2 score landscapes for C1–C5. Left panels (A, C, E, G, I, and K) show the PCA score distributions for each class, while the corresponding right panels (B, D, F, H, J, and L) display the spatial distribution of PC1–PC2 scores as false-colour landscapes. The colour scale represents the PC1 score, illustrating the variation and spatial heterogeneity captured by the principal components across the five classes. C1: orientin–BChE; C2: 3,5-diferuloylquinic acid–BChE; C3: orientin–AChE; C4: 3,5-diferuloylquinic acid–amylase; and C5: orientin–glucosidase.

The FEL analysis provides complementary information by identifying preferred conformational basins and transitions between them. Distinct low-energy basins indicate frequently populated conformational substates, whereas multiple separated basins may reflect transitions between alternative states. The integration of PCA with FEL analysis is therefore useful for characterizing conformational heterogeneity and identifying representative states from MD trajectories.^48^

In summary, the PCA/FEL results support a relatively restricted conformational ensemble for C1–C3, increased collective dynamics for C4, and pronounced conformational heterogeneity for C5. This interpretation is consistent with the RMSD/RMSF results, particularly the enhanced flexibility observed for C5. Nevertheless, PCA/FEL should be considered a complementary analysis rather than standalone evidence of complex stability or binding affinity, because Cartesian PCA can also be influenced by global or collective motions and by the quality and extent of trajectory sampling.^47,49^

## Conclusion

This study provides a comprehensive solvent-resolved comparison of the phytochemical composition and *in vitro* biological properties of *Achillea goniocephala* and *Achillea brachyphylla*. LC-MS-QTOF profiling revealed a chemically diverse metabolome comprising more than 90 tentatively annotated constituents, with acylquinic acids, phenolic acids, flavonoid glycosides, methoxylated flavones and terpenoid-related metabolites representing major components of the chemical profiles. Multivariate analysis demonstrated pronounced differentiation among the species – extract combinations, while the distribution of several tentatively annotated metabolites revealed species-associated qualitative signatures. In particular, achillin/matricin- and achimilic-acid-related features were characteristic of *A. goniocephala*, whereas deoxyloganin and verbascoside were among the features associated with *A. brachyphylla*. These differences highlight the potential value of such compounds as candidate species-discriminating markers, although targeted confirmation remains necessary. Extraction solvent strongly influenced both chemical composition and biological performance, but no single solvent was optimal for all measured responses. Polar extracts generally favoured phenolic recovery, radical-scavenging activity and reducing capacity, with *A. goniocephala* showing particularly pronounced CUPRAC and FRAP responses. In contrast, metal-chelating and phosphomolybdenum activities followed partly different patterns. Enzyme inhibition was similarly target-dependent: *A. brachyphylla* showed notable BChE and α-amylase inhibition in less polar fractions, *A. goniocephala* exhibited comparatively strong α-glucosidase activity in several medium-to-polar extracts, whereas ethanol and ethanol/water extracts of both species showed the strongest tyrosinase inhibition. Molecular docking further identified favourable interactions between selected phytochemicals and the investigated enzyme targets, while molecular dynamics simulations supported the stability of representative ligand–protein complexes, providing complementary structural context for the observed enzyme inhibitory activities. These contrasting responses demonstrate that the biological activity cannot be inferred from extract polarity, TPC or TFC alone, and instead, it reflects the distinct chemical composition of each fraction. Overall, the solvent-resolved approach revealed marked chemical and functional diversities both between the two *Achillea* species and among extracts of the same species, underscoring the limitations of characterising complex botanical materials through a single extraction system. The present findings broaden the available phytochemical and bioactivity data for these comparatively understudied taxa, particularly *A. brachyphylla*, and provide a rational basis for selecting specific extracts for future phytochemical and functional investigations. Nevertheless, the LC-MS assignments remain predominantly tentative and the biological effects were established using *in vitro* assays; therefore, targeted metabolite confirmation, quantitative analysis, bioactivity-guided fractionation and appropriate mechanistic studies will be required to identify the constituents underlying the most prominent activities and to establish their broader biological relevance.

## Author contributions

Conceptualization: MT, AK, VA, MVC, AIU, GZ; methodology: MT, AK, VA, MVC, AIU, GA, ES; software: MVC, AIU; validation: GZ; formal analysis: GZ; investigation: MT, AK, NPB, MMB, GA, ES, SY, GZ; resources: NPB, MMB; data curation: MT, AK, GZ; writing – original draft preparation: MT, AK, VA, MVC, AIU, GZ; writing—review and editing: NPB, MMB, ES, GA, SY; visualization: MVC, AIU; supervision: GZ; project administration: GZ; funding acquisition: GZ. All authors have read and agreed to the published version of the manuscript.

## Conflicts of interest

There are no conflict to declare.

## Abbreviations

AB: *Achillea brachyphylla*
ABTS: 2,2′-Azino-bis(3-ethylbenzothiazoline-6-sulfonic acid)
ACAE: Acarbose equivalents
AChE: Acetylcholinesterase
AG: *Achillea goniocephala*
ANOVA: Analysis of variance
BChE: Butyrylcholinesterase
CUPRAC: Cupric reducing antioxidant capacity
DPPH: 2,2-Diphenyl-1-picrylhydrazyl
E: Ethanol
EA: Ethyl acetate
EDTA: Ethylenediaminetetraacetic acid
ESI: Electrospray ionization
E_W: Ethanol/water
FEL: Free-energy landscape
FRAP: Ferric reducing antioxidant power
GAE: Gallic acid equivalents
GALAE: Galantamine equivalents
GROMACS: GROningen MAchine for chemical simulations
H: *n*-Hexane
KAE: Kojic acid equivalents
LC-MS-QTOF: Liquid chromatography-quadrupole time-of-flight mass spectrometry
LINCS: Linear constraint solver
MC: Metal-chelating activity
MD: Molecular dynamics
MMFF94: Merck molecular force field 94
MS: Mass spectrometry
MS/MS: Tandem mass spectrometry
*m*/*z*: Mass-to-charge ratio
NPT: Constant number of particles, pressure and temperature ensemble
NVT: Constant number of particles, volume and temperature ensemble
PBD: Phosphomolybdenum assay
PCA: Principal component analysis
PDBQT: Protein Data Bank, partial charge (Q), and atom type (T) format
PERMANOVA: Permutational multivariate analysis of variance
PLIP: Protein–Ligand interaction profiler
PLS-DA: Partial least squares discriminant analysis
PME: Particle-mesh Ewald
POCASA: Pocket-cavity search application
RE: Rutin equivalents
RMSD: Root-mean-square deviation
RMSF: Root-mean-square fluctuation
RT: Retention time
SASA: Solvent-accessible surface area
SD: Standard deviation
SDF: Structure-data file
SMILES: Simplified molecular-input line-entry system
TE: Trolox equivalents
TFC: Total flavonoid content
TIP3P: Transferable intermolecular potential with three points
TPC: Total phenolic content
UHPLC: Ultra-high-performance liquid chromatography
VIP: Variable importance in projection
W: Water
na: Not active

## Supplementary Material

RA-OLF-D6RA08354D-s001

## Data Availability

Data will be made available on request to authors. Supplementary information (SI) is available. See DOI: https://doi.org/10.1039/d6ra08354d.
