# Supplementary material for: Phytochemistry, bioactivity, and in silico studies on two understudied Achillea species (A. goniocephala and A. brachyphylla): species- and solvent-dependent patterns
Source: RSC Adv. 2026 Oct 2;16(46):59710–32. doi: 10.1039/d6ra08354d (PMC13631748; doi:10.1039/d6ra08354d)
Supplement: Supplementary file 1 [file RA-016-D6RA08354D-s001.pdf]

Supplementary Information (SI) for RSC Advances.
This journal is © The Royal Society of Chemistry

Table S1. Grid center and size used for docking the most abundant phytochemicals in the extracts into the selected enzyme and protein targets.

Target Protein	PDB ID	Center X, Y, Z (Å)	Grid Size X, Y, Z (Å)
AChE	7E3H	-54.44, 32.90, -28.65	25, 25, 25
BChE	6EQP	32.16, -16.33, 40.73	25, 25, 25
α-amylase	2QV4	14.188, 48.964, 22.886	28, 28, 24
α-glucosidase	7KBJ	-18.79, 2.18, 15.75	25, 25, 25
Tyrosinase	6QXD	21.81, 12.22, 91.40	25, 25, 25

Table S2. Binding energy of the most abundant compounds in the extracts of *Achillea goniocephala* and *Achillea brachyphylla* against selected enzymes.

Compound	Target	PDB ID	Binding energy (kcal/mol)	RMSD (Å)	Type of interaction and Binding site residues
Orientin	BChE	6EQP	-9.9	0.41	Hbond: ASP:70;ASP:70;TRP:82;THR:120;TRP:430;HIS:438;HIS:438<br>Hydrophobic: ASP:70<br>Pi-stacking: PHE:329
3,5-diferuloylquinic acid	BChE	6EQP	-9.6	1.436	Hbond: TRP:82;TYR:128;LEU:286;TRP:430<br>Hydrophobic: TRP:82;TRP:82;ALA:328;PHE:329;PHE:329;PHE:398;TRP:430<br>Pi-stacking: TRP:231;TRP:231<br>Salt bridge: HIS:438
Verbasoside	BChE	6EQP	-9.4	7.132	Hbond: TRP:82;GLY:117;TYR:128;TYR:128;SER:198;SER:198;TRP:430<br>Hydrophobic: TRP:82;TRP:82
Orientin	AChE	7E3H	-9.4	0.984	Hbond: GLY:120;GLY:121;GLU:202;TYR:337;HIS:447<br>Hydrophobic: TRP:86;TYR:337;TYR:341
3,5-diferuloylquinic acid	Amylase	2qv4	-9.2	1.11	Hbond: GLN:63;GLN:63;THR:163;HIS:201;ASP:300;HIS:305<br>Hydrophobic: TRP:59;TRP:59;TRP:59<br>Salt bridge: HIS:299;HIS:305
Orientin	Glucosidase	7KBJ	-8.8	0.049	Hbond: LEU:252;GLY:253;GLY:254;MET:289;TYR:291;TYR:291;ARG:334;ARG:352;ARG:352<br>Hydrophobic: PHE:350;PHE:350<br>Pi-cation: ARG:352
Chlorogenic acid	AChE	7E3H	-8.8	0.328	Hbond: TYR:124;GLU:202;PHE:295;TYR:337<br>Hydrophobic: TRP:86;TRP:86;TRP:86;TYR:124;PHE:297;TYR:341
3,4-diferuloylquinic acid	BChE	6EQP	-8.6	0.212	Hbond: ASP:70;ASP:70;TRP:82;ASN:83;GLY:117;SER:198;LEU:286;TRP:430;TYR:440<br>Hydrophobic: TRP:82;ALA:328;PHE:329;PHE:329;TYR:332;TRP:430<br>Salt bridge: HIS:438
Achimilic acid	AChE	7E3H	-8.6	0.554	Hbond: GLY:121;GLY:122;TYR:124;TYR:124;TYR:337;HIS:447<br>Hydrophobic: TRP:86;TRP:86;TRP:86;TYR:124;PHE:295;TYR:337;PHE:338;PHE:338
Verbasoside	Amylase	2qv4	-8.6	1.094	Hbond: TYR:62;HIS:101;ARG:195;ARG:195;LYS:200;HIS:201;I

Compound	Target	PDB ID	Binding energy (kcal/mol)	RMSD (Å)	Type of interaction and Binding site residues
					LE:235;HIS:299;ASP:300;ASP:300;HIS:305<br>Hydrophobic: LEU:162;LEU:165<br>Pi-stacking: HIS:201
3,4-diferuloylquinic acid	Glucosidase	7KBJ	-8.5	0.894	Hbond: GLY:253;GLY:253;LYS:283;TYR:291;ALA:345;TYR:348<br>Hydrophobic: LEU:252;LEU:344;PHE:350<br>Salt bridge: ARG:352;ARG:352
Chlorogenic acid	BChE	6EQP	-8.5	0.767	Hbond: ASP:70;HIS:438<br>Hydrophobic: PHE:329;PHE:398<br>Pi-stacking: TRP:231;TRP:231;PHE:329<br>Salt bridge: HIS:438
Cirsiliol	BChE	6EQP	-8.5	0.636	Hbond: GLY:78;TRP:82;GLY:117;SER:198;PRO:285;TYR:332;TRP:430<br>Hydrophobic: PHE:329;PHE:329
Cirsiliol	Amylase	2qv4	-8.4	0.929	Hbond: GLN:63;ARG:195;ARG:195;GLU:233;GLU:233;HIS:299<br>Hydrophobic: TRP:58;TRP:59;TRP:59;TYR:62<br>Pi-stacking: TRP:59;TYR:62
Orientin	Amylase	2qv4	-8.3	0.92	Hbond: GLN:63;TYR:151;ARG:195;HIS:201;HIS:299<br>Hydrophobic: TRP:58;TYR:62;LEU:162
Chlorogenic acid	Glucosidase	7KBJ	-8.3	2.573	Hbond: LYS:283;SER:290;TYR:291;LEU:344;PHE:350;ARG:352<br>Hydrophobic: TYR:291;PHE:350
Verbasoside	AChE	7E3H	-8.3	1.019	Hbond: GLN:71;ASP:74;ASP:74;GLY:120;GLY:120;GLY:121;GLY:121;TYR:124;TYR:133;SER:203;TYR:337<br>Hydrophobic: TRP:86;TRP:86;TRP:86;TYR:337<br>Pi-stacking: TRP:86
Verbasoside	Glucosidase	7KBJ	-8.1	0.509	Hbond: GLN:247;GLU:248;GLU:248;GLY:253;GLY:254;LYS:283;PHE:350;PHE:350<br>Hydrophobic: LEU:252;TRP:331<br>Salt bridge: ARG:352;ARG:352<br>Pi-cation: ARG:352
Eupatorin	Glucosidase	7KBJ	-8.1	0.596	Hbond: GLY:253<br>Hydrophobic: TYR:291;TYR:291;TYR:291;TRP:331
3,4-diferuloylquinic acid	Amylase	2qv4	-8.1	1.055	Hbond: GLN:63;THR:163;THR:163;ARG:195;GLU:233;HIS:299<br>Hydrophobic: TRP:59;TRP:59;TYR:62;TYR:62<br>Salt bridge: HIS:305
3,5-diferuloylquinic acid	Glucosidase	7KBJ	-8	3.581	Hbond: LYS:283;MET:289;TYR:291;TYR:348;ARG:352;ARG:352<br>Hydrophobic: LEU:252;TRP:331;LEU:344<br>Salt bridge: ARG:352<br>Pi-cation: ARG:334
Cirsiliol	Glucosidase	7KBJ	-8	0.752	Hbond: ALA:345;TYR:348;ARG:352<br>Hydrophobic: TYR:291;LEU:344;PHE:350

Compound	Target	PDB ID	Binding energy (kcal/mol)	RMSD (Å)	Type of interaction and Binding site residues
Eupatorin	Amylase	2qv4	-7.9	1.082	Hbond: GLN:63;HIS:299;ASP:300<br>Hydrophobic: TRP:58;TRP:59;TYR:62<br>Pi-stacking: TRP:59;TRP:59
Chlorogenic acid	Amylase	2qv4	-7.6	8.088	Hbond: GLN:63;ARG:195;GLU:233;GLU:233<br>Hydrophobic: TRP:58;TRP:59;TYR:62;LEU:165<br>Pi-stacking: TYR:62
Achimilic acid	BChE	6EQP	-7.6	4.494	Hydrophobic:<br>TRP:82;TRP:82;TRP:82;LEU:125;ALA:328;TYR:332<br>Salt bridge: HIS:438
Cirsiliol	AChE	7E3H	-7.5	0.521	Hbond: GLY:120;TYR:133;GLU:202;TYR:337<br>Hydrophobic: TRP:86;TRP:86;PHE:338
Artemetin	Glucosidase	7KBJ	-7.4	0.346	Hbond: ALA:345;ARG:352<br>Hydrophobic: TYR:291;TYR:291<br>Pi-cation: ARG:352
Artemetin	BChE	6EQP	-7.3	0.018	Hbond:<br>ASP:70;TRP:82;GLY:116;GLY:117;SER:198;SER:198;TRP:430<br>Pi-stacking: PHE:329
Artemetin	Amylase	2qv4	-7.3	0.041	Hbond: LYS:200;HIS:201<br>Hydrophobic: LEU:162
Artemetin	AChE	7E3H	-7.3	0.067	Hbond: GLY:126;TYR:337<br>Hydrophobic: TRP:86;TRP:86;PHE:297;PHE:338
Achimilic acid	Amylase	2qv4	-7.2	0.359	Hbond: GLN:63;HIS:101;ASP:300<br>Hydrophobic: TRP:59;TYR:62;TYR:62;LEU:162<br>Salt bridge: HIS:305
Eupatorin	AChE	7E3H	-7	0.768	Hbond: GLY:120;TYR:133;TYR:337<br>Hydrophobic: TRP:86;PHE:338
Eupatorin	BChE	6EQP	-6.7	1.064	Hbond: TRP:82;THR:120;THR:120;TRP:430<br>Hydrophobic: TRP:430
Achimilic acid	Glucosidase	7KBJ	-6.6	7.74	Hbond: LYS:283;TYR:291;ARG:352<br>Hydrophobic: LEU:252;TYR:291<br>Salt bridge: ARG:352
3,5-diferuloylquinic acid	Tyrosinase	6QXD	-6.2	4.162	Hbond: ASN:57;VAL:218<br>Hydrophobic: HIS:208;VAL:218;ALA:221<br>Pi-stacking: HIS:208<br>Salt bridge: ARG:209
3,4-diferuloylquinic acid	AChE	7E3H	-6.1	1.016	Hbond:<br>THR:83;TRP:86;GLY:120;GLY:121;GLY:122;TYR:124;ALA:127;TYR:133;GLU:202;SER:203<br>Hydrophobic:<br>ASP:74;TRP:86;TRP:86;TRP:86;LEU:130;PHE:338;TYR:341<br>Salt bridge: HIS:447
Verbasoside	Tyrosinase	6QXD	-6.1	1.046	Hbond: ASN:57;ASN:205;MET:215<br>Hydrophobic: PHE:197;VAL:218;VAL:218
Chlorogenic acid	Tyrosinase	6QXD	-5.9	0.499	Hbond: MET:215<br>Hydrophobic: HIS:208;VAL:218<br>Pi-stacking: HIS:208<br>Salt bridge: ARG:209;ARG:209
Achimilic acid	Tyrosinase	6QXD	-5.7	1.424	Hbond: ARG:209;VAL:218<br>Hydrophobic: ASN:205;HIS:208;VAL:218
3,5-diferuloylquinic acid	AChE	7E3H	-5.6	0.549	Hbond: TYR:72;TYR:337;TYR:341<br>Hydrophobic:

Compound	Target	PDB ID	Binding energy (kcal/mol)	RMSD (Å)	Type of interaction and Binding site residues
					ASP:74;TRP:86;TRP:86;TYR:124;PHE:297;PHE:338;TYR:341
Cirsiliol	Tyrosinase	6QXD	-5.5	0.832	Hbond: HIS:42<br>Hydrophobic: VAL:218<br>Pi-stacking: HIS:208
3,4-diferuloylquinic acid	Tyrosinase	6QXD	-5.4	0.849	Hbond: GLY:216<br>Hydrophobic: ASN:205;HIS:208;VAL:217<br>Salt bridge: HIS:208;ARG:209;ARG:209
Artemetin	Tyrosinase	6QXD	-5.3	0.047	Hbond: ARG:209;ARG:209<br>Hydrophobic: VAL:218
Eupatorin	Tyrosinase	6QXD	-5.1	0.862	Hydrophobic: VAL:218
Orientin	Tyrosinase	6QXD	-3.9	1.139	Hydrophobic: VAL:218<br>Pi-stacking: HIS:208
